# Supplementary material for: NorQD AAA+ complex drives metal insertion by a twisting mechanism
Source: Nat Commun. 2026 Mar 27;17:3032. doi: 10.1038/s41467-026-71044-4 (PMC13036017; doi:10.1038/s41467-026-71044-4)
Supplement: Supplementary file 1 — Supplementary Information [file 41467_2026_71044_MOESM1_ESM.pdf]

Supplementary Information for

NorQD AAA+ complex drives metal insertion by a twisting mechanism

Maximilian Kahle[1,2&], Sofia Appelgren[2,3&], Finja König[2], Marta Carroni[2,4], Pia Ädelroth[2*], Petra Wendler[1*]

[1] Institute of Biochemistry and Biology, Department of Biochemistry, University of Potsdam, Karl-Liebknecht Strasse 24-25, 14476 Potsdam-Golm, Germany.

[2] Department of Biochemistry and Biophysics, Stockholm University, Svante Arrhenius väg 16C, 11691 Stockholm, Sweden.

[3] Department of Biology, Philipps University Marburg, 35043 Marburg, Germany

[4] Swedish Cryo-EM Facility, Science for Life Laboratory Stockholm University, Solna, Sweden

[&]These authors contributed equally

[*]Correspondence should be addressed to Pia Ädelroth (pia.adelroth@dbb.su.se) and Petra Wendler (petra.wendler@uni-potsdam.de).

Supplementary Figures

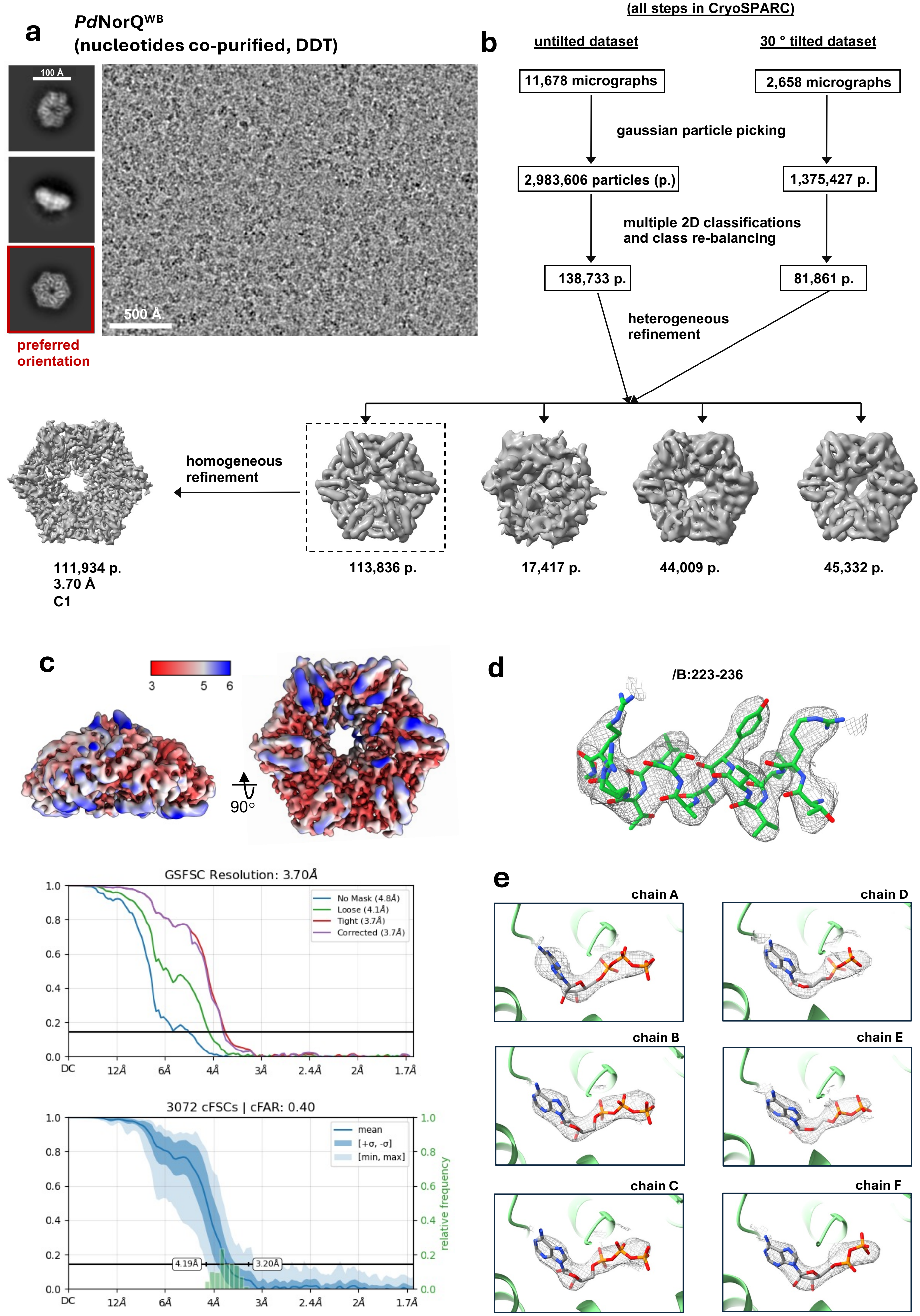

Supplementary Figure 1 | Cryo-EM data processing and model building for *Pd*Q^{WB}. a, Representative micrograph and 2D classes. **b**, Cryo-EM processing workflow. **c**, Local resolution map, 3D Fourier shell correlation (FSC) curve and conical FSC (cFSC) curve. **d**, EM density and atomic model of one NorQ α-helix (aa223-236) in chain B. **e**, EM densities and atomic models of the nucleotides in the binding pockets of NorQ.

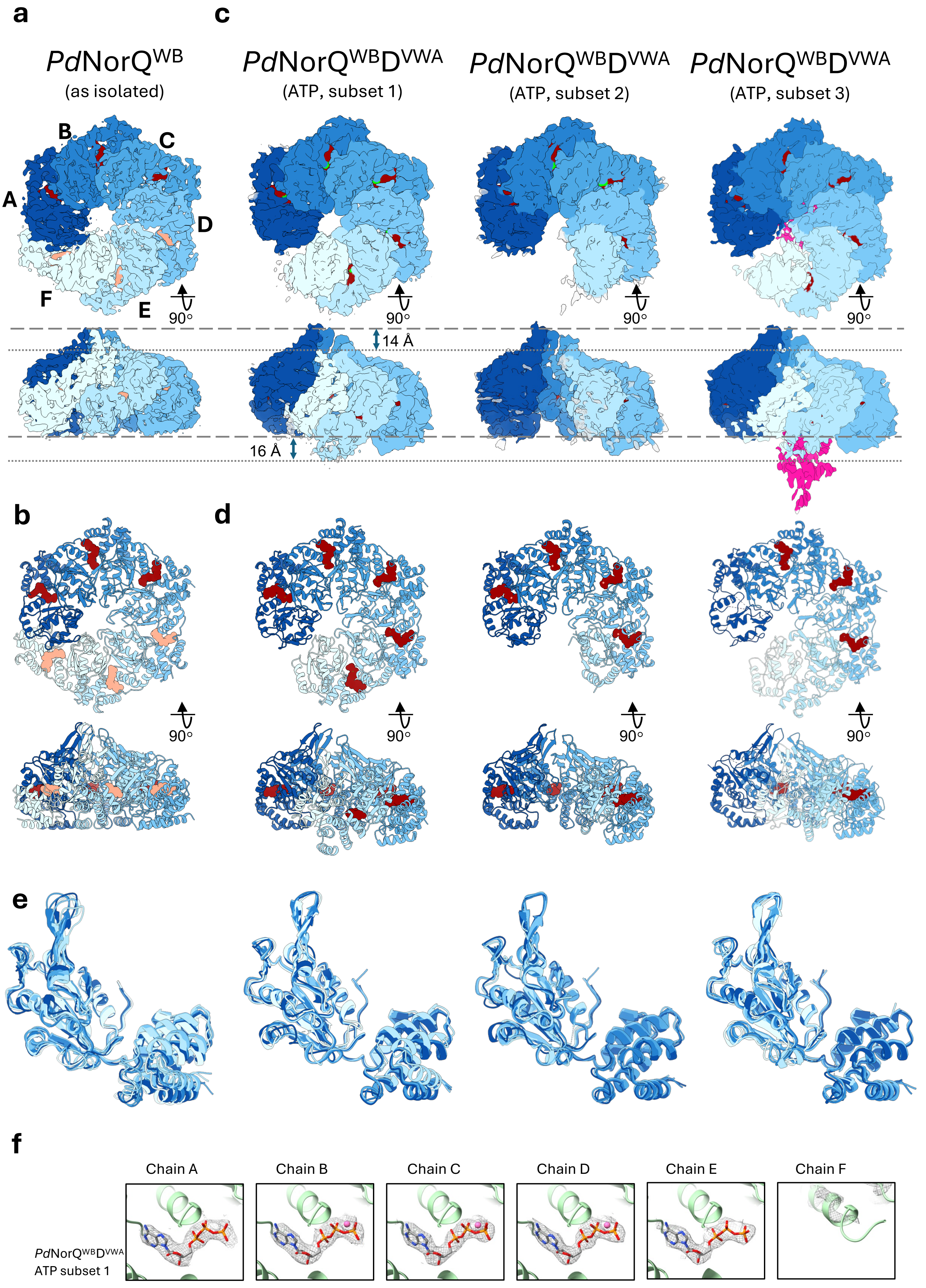
a
c
PdNorQWB
(as isolated)
PdNorQWBDVWA
(ATP, subset 1)
PdNorQWBDVWA
(ATP, subset 2)
PdNorQWBDVWA
(ATP, subset 3)
A
B
C
D
E
F
90°
14 Å
16 Å
b
d
e
f
Chain A
Chain B
Chain C
Chain D
Chain E
Chain F
PdNorQWBDVWA
ATP subset 1

Supplementary Figure 2 | Structures of *Pd*NorQ^{WB} and *Pd*NorQ^{WB}D^{VWA}. The models depicted in insets **a-d** are aligned on NorQ subunit A. **a**, Cryo-EM map of *Pd*NorQ^{WB} shown as top view (N-terminal side of NorQ, top row) and side view (bottom row). The individual subunits of NorQ are colour coded in shades of blue according to their position in the spiral, from top (A, dark) to bottom (F, light). Density for ATP and ADP is shown in dark red and peach, respectively. **b,** PDB model derived from the map shown in a. **c,** Cryo-EM maps of *Pd*NorQ^{WB}D^{VWA} shown as top view (top row) and side view (bottom row). Subunits are colour coded as in a. Residual density for the VWA domain of NorD is coloured pink. The dashed line indicates the position of subunit A of NorQ in the spiral, on which all complexes have been superimposed. **d,** PDB models derived from the maps shown in c. **e,** Superposition of all AAA+ domains of NorQ of the complexes shown in b and d on the N-terminal, Rossman-type fold subdomains. **f,** EM densities around the nucleotide in the binding pockets of NorQ subunits in *Pd*NorQ^{WB}D^{VWA} plus ATP, subset 1. Although the orientation of the small AAA+ subdomain of this state in c indicates a post-hydrolysis conformation, the density in chain E resembles that of ATP, possibly indicating bound ADPP$_i$ in this binding pocket.

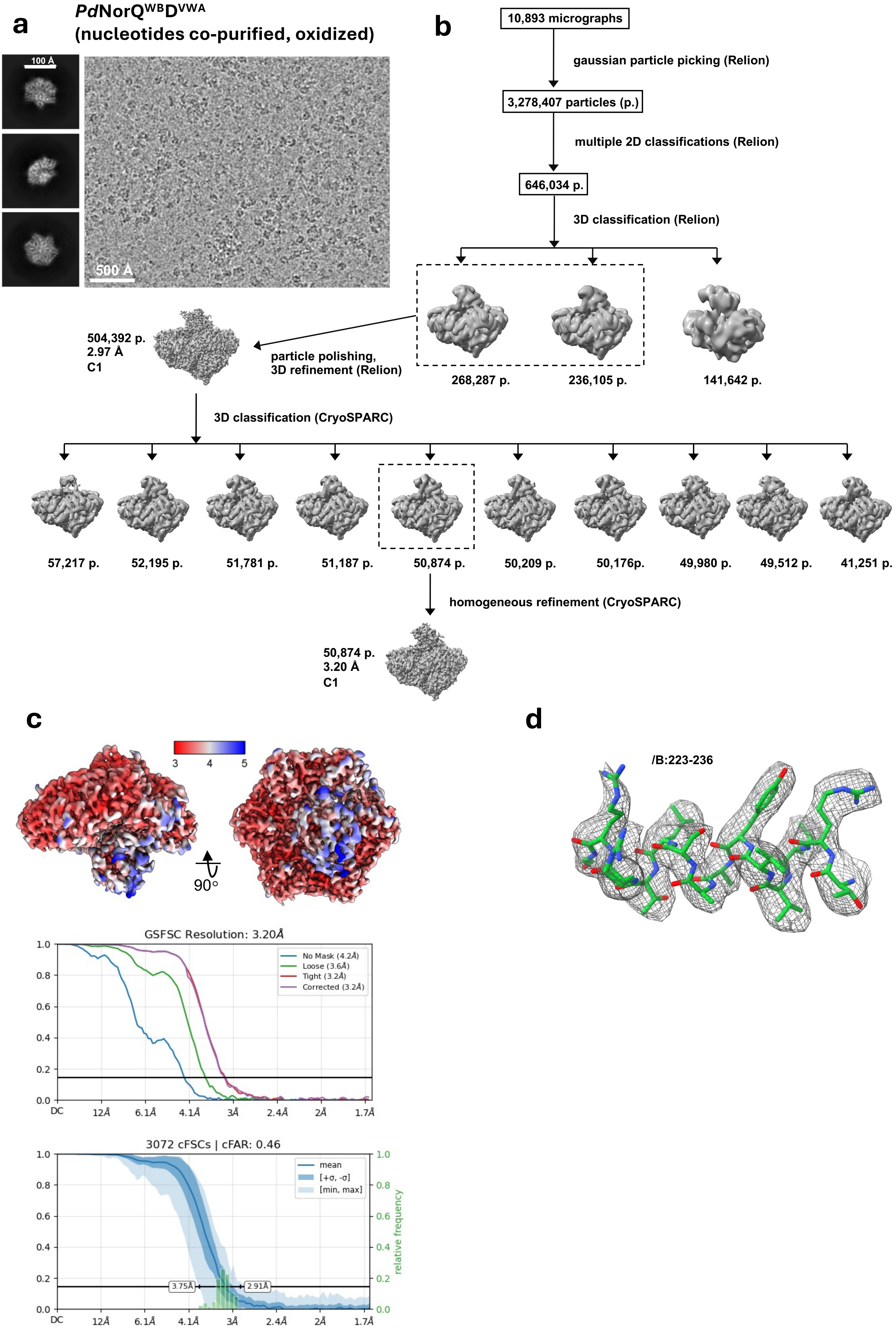
a
PdNorQ^{WB}D^{VWA}
(nucleotides co-purified, oxidized)
100 Å
500 Å
b
10,893 micrographs
gaussian particle picking (Relion)
3,278,407 particles (p.)
multiple 2D classifications (Relion)
646,034 p.
3D classification (Relion)
268,287 p.
236,105 p.
141,642 p.
particle polishing,
3D refinement (Relion)
504,392 p.
2.97 Å
C1
3D classification (CryoSPARC)
57,217 p.
52,195 p.
51,781 p.
51,187 p.
50,874 p.
50,209 p.
50,176p.
49,980 p.
49,512 p.
41,251 p.
homogeneous refinement (CryoSPARC)
50,874 p.
3.20 Å
C1
c
3
4
5
90°
GSFSC Resolution: 3.20Å
No Mask (4.2Å)
Loose (3.6Å)
Tight (3.2Å)
Corrected (3.2Å)
1.0
0.8
0.6
0.4
0.2
0.0
DC
12Å
6.1Å
4.1Å
3Å
2.4Å
2Å
1.7Å
3072 cFSCs | cFAR: 0.46
mean
[+σ, -σ]
[min, max]
relative frequency
3.75Å
2.91Å
d
/B:223-236

Supplementary Figure 3 | Cryo-EM data processing and model building for *Pd*NorQ^{WB}D^{VWA}. a, Representative micrograph and 2D classes. **b**, Cryo-EM processing workflow. **c**, Local resolution map, 3D Fourier shell correlation (FSC) curve and conical FSC (cFSC) curve. **d**, EM density and atomic model of one NorQ α-helix (aa223-236) in chain B.

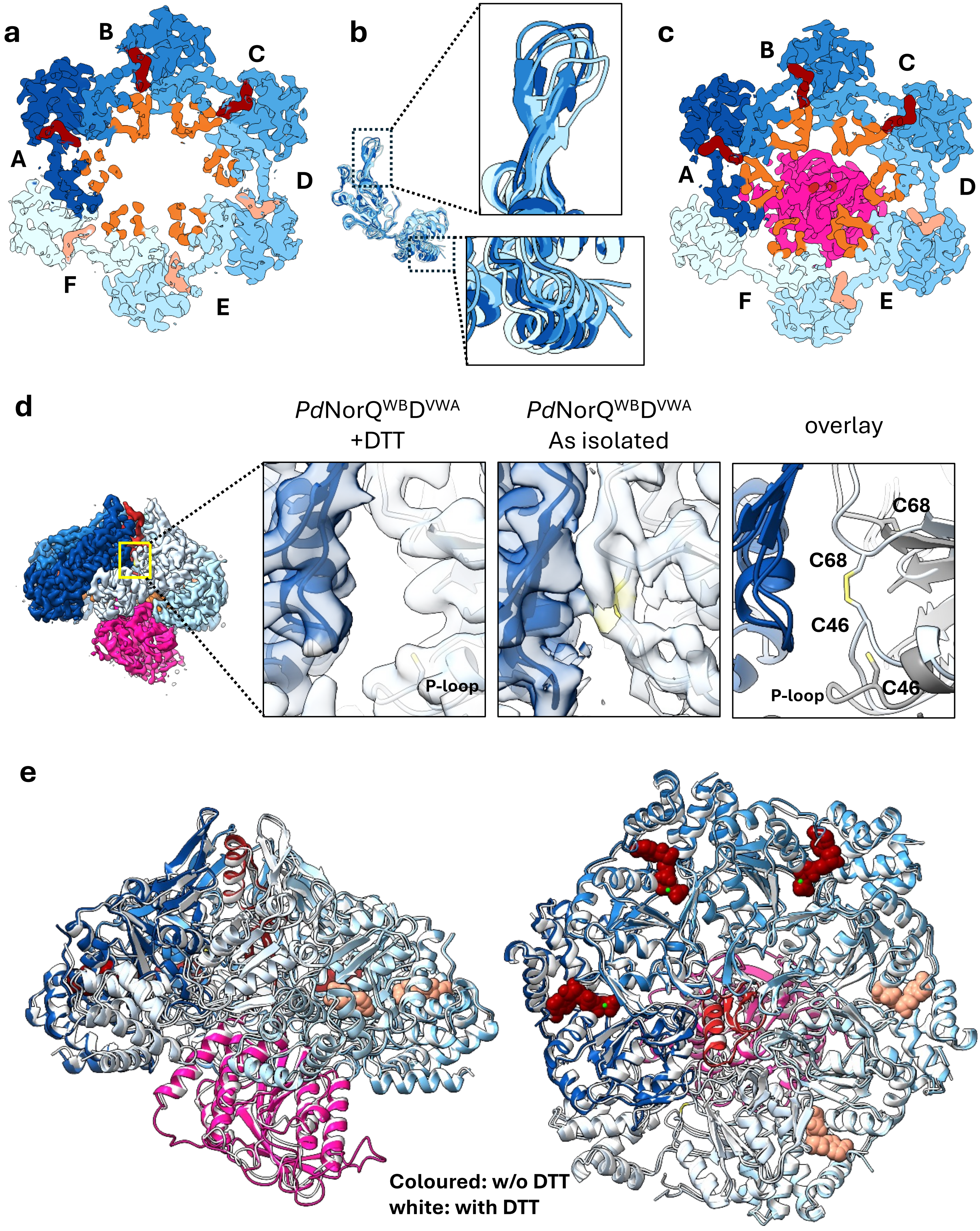
a
B
C
A
D
F
E
b
c
B
C
A
D
F
E
d
PdNorQWBDVWA
+DTT
PdNorQWBDVWA
As isolated
overlay
P-loop
C68
C68
C46
P-loop
C46
e
Coloured: w/o DTT
white: with DTT

Supplementary Figure 4 | Effects of the interaction between *Pd*NorD^{VWA} and *Pd*NorQ^{WB}. a, Top view representation of isolated cryo-EM densities of the *Pd*NorQ^{WB} map around residues 148- 270 and the nucleotide, including the postS1 loops (orange) and the α-helical subdomain of the AAA+ proteins. The individual NorQ subunits are colour-coded and named according to Figure 1. **b,** Superposition of all AAA+ domains of the *Pd*NorQ^{WB}D^{VWA} complex on the N-terminal, Rossman-type fold subdomains. Enlargements of the H2i region and the α-helical subdomain are shown. **c,** Isolated cryo-EM densities of the oxidized, as-isolated *Pd*NorQ^{WB}D^{VWA} map around residues 148- 270 of NorQ, the nucleotide, and the NorD^{VWA} domain. **d,** Comparison of cryo-EM maps of *Pd*NorQ^{WB}D^{VWA} in the presence and absence of 10 mM DTT. Section of the EM map with fitted model around the nucleotide binding P-loop of the nucleotide-free seam subunit of the *Pd*NorQ^{WB}D complex in the presence of 10 mM DTT (left) and in the oxidized, as isolated complex (middle). The two complexes are superimposed on all AAA+ domains. Superimposition of the models with the reduced seam subunit in grey and the oxidized, as-isolated seam subunit with the disulfide bridge between C46 and C68 in light blue (right). **e,** Superimposition of the reduced (white) and oxidized (coloured) models on all AAA+ domains. RMSD between 1479 pruned Cα atom pairs is 0.839 Å (across all 1525 Cα atom pairs: 1.075 Å).

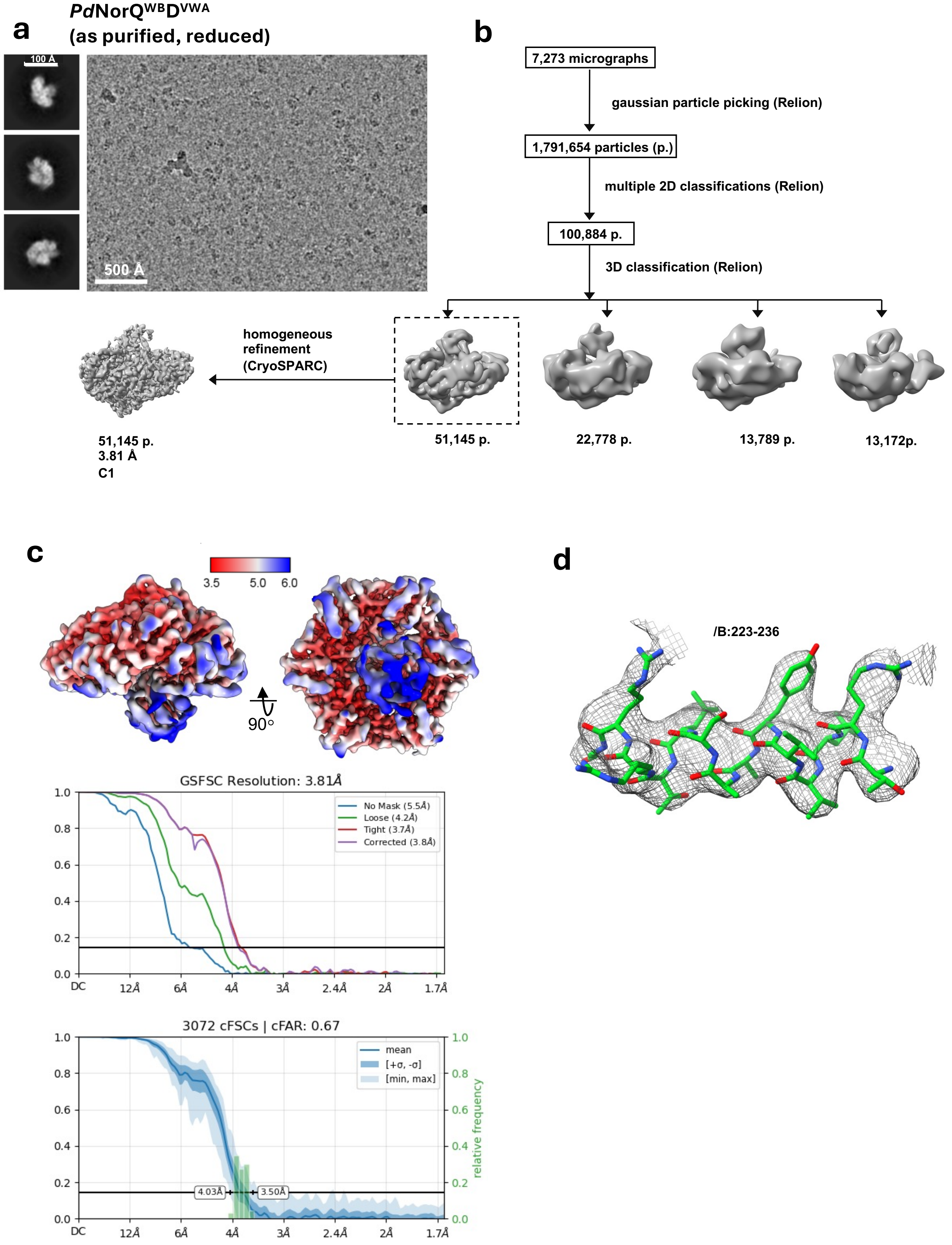

Supplementary Figure 5 | Cryo-EM data processing and model building for reduced *Pd*NorQ^{WB}D^{VWA}. a, Representative micrograph and 2D classes. **b**, Cryo-EM processing workflow. **c**, Local resolution map, 3D Fourier shell correlation (FSC) curve and conical FSC (cFSC) curve **d,** EM density and atomic model of one NorQ α-helix (aa223-236) in chain B.

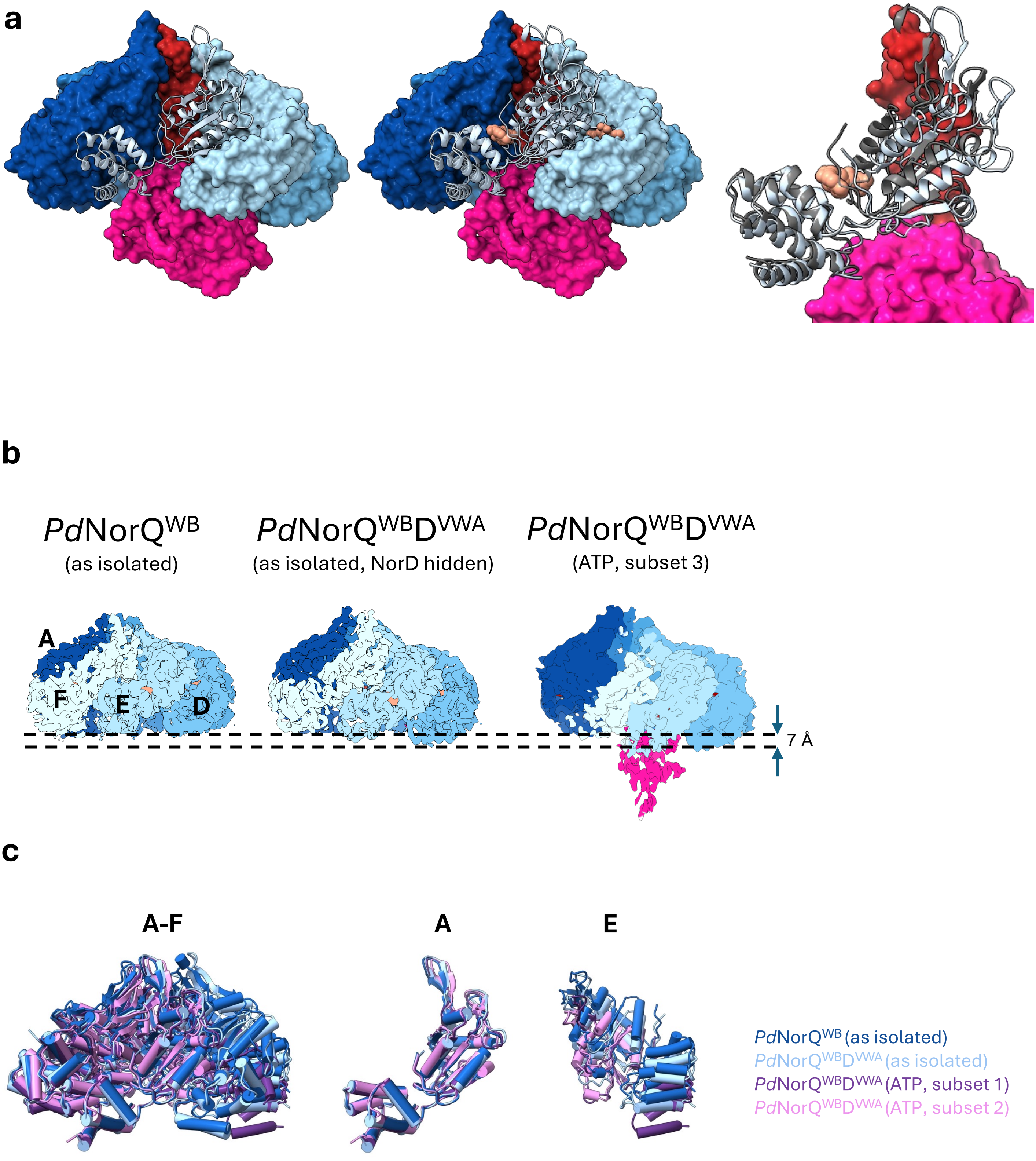

Supplementary Figure 6 | Intercalation of *Pd*NorD^{finger} at *Pd*NorQ^{WB} seam and induced spiral staircase geometry. a, left: Side-view representation of the *Pd*NorQ^{WB}D^{VWA} model shown as surface, except for the seam subunit of NorQ, which is shown as ribbon model. Middle: Superposition of models of *Pd*NorQ^{WB} and *Pd*NorQ^{WB}D^{VWA} on topmost ATP bound AAA+ domain of NorQ. *Pd*NorQ^{WB}D^{VWA} is shown as seen on the left, while only seam subunit of *Pd*NorQ^{WB} is shown. Right: Close-up view of seam subunits of *Pd*NorQ^{WB} and *Pd*NorQ^{WB}D^{VWA} models as seen in the middle, but the remaining AAA+ subunits of NorQ are hidden. **b,** Cryo-EM maps of specified models, superimposed on subunit A of NorQ and shown as side-views. The 7Å increase in total helical rise over subunits A-D is indicated. **c,** Overlay of the indicated models to visualise change in helical rise. All models are superimposed on NorQ subunit A.

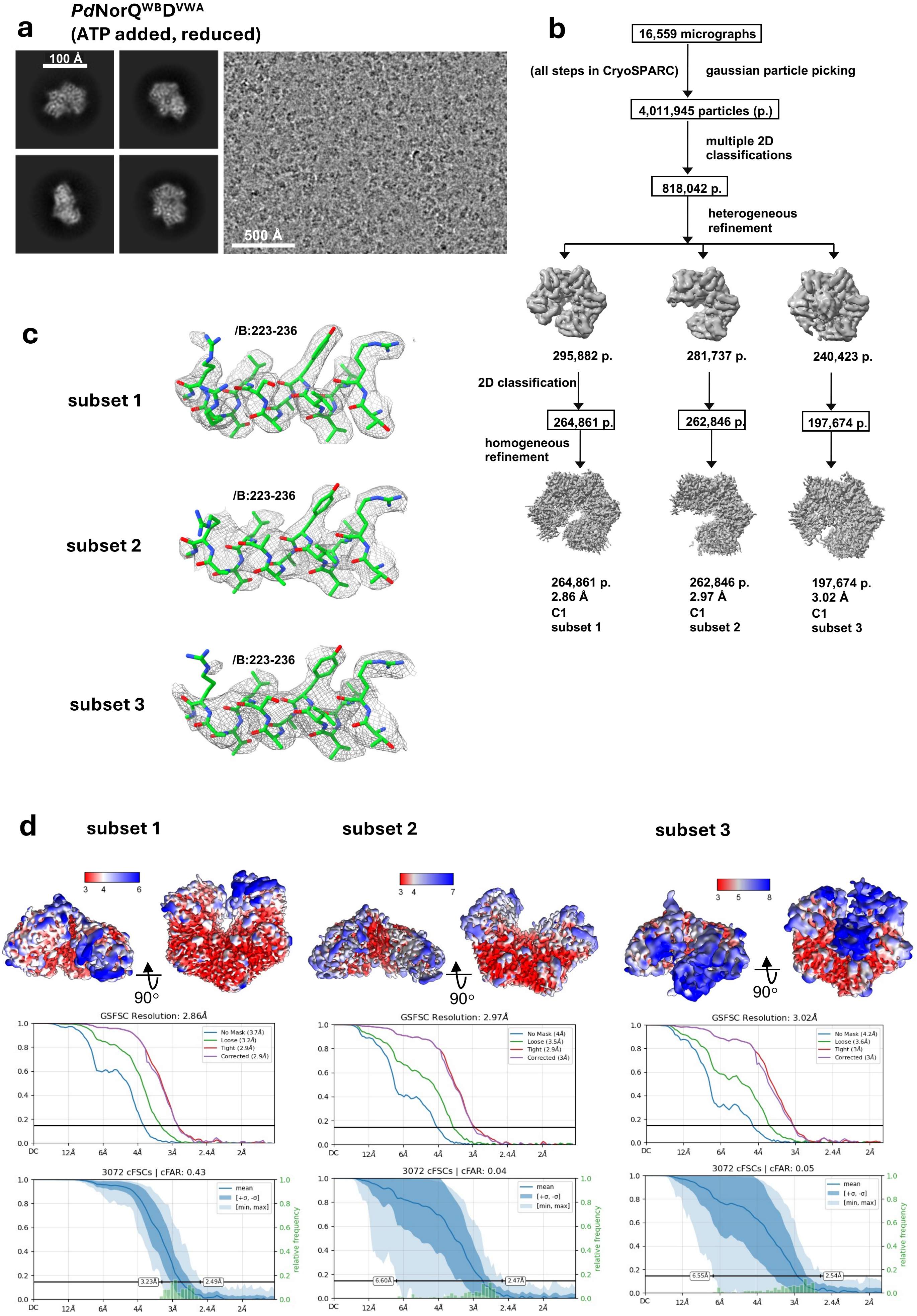

a
PdNorQ^WB D^VWA
(ATP added, reduced)
100 Å
500 Å
b
16,559 micrographs
(all steps in CryoSPARC)
gaussian particle picking
4,011,945 particles (p.)
multiple 2D classifications
818,042 p.
heterogeneous refinement
295,882 p.
281,737 p.
240,423 p.
2D classification
264,861 p.
262,846 p.
197,674 p.
homogeneous refinement
264,861 p.
2.86 Å
C1
subset 1
262,846 p.
2.97 Å
C1
subset 2
197,674 p.
3.02 Å
C1
subset 3
c
subset 1
/B:223-236
subset 2
/B:223-236
subset 3
/B:223-236
d
subset 1
subset 2
subset 3
90°
GSFSC Resolution: 2.86Å
No Mask (3.7Å)
Loose (3.2Å)
Tight (2.9Å)
Corrected (2.9Å)
GSFSC Resolution: 2.97Å
No Mask (4Å)
Loose (3.5Å)
Tight (2.9Å)
Corrected (3Å)
GSFSC Resolution: 3.02Å
No Mask (4.2Å)
Loose (3.6Å)
Tight (3Å)
Corrected (3Å)
3072 cFSCs | cFAR: 0.43
3072 cFSCs | cFAR: 0.04
3072 cFSCs | cFAR: 0.05
mean
[+σ, -σ]
[min, max]
relative frequency
3.23Å
2.49Å
6.60Å
2.47Å
6.55Å
2.54Å
DC
12Å
6Å
4Å
3Å
2.4Å
2Å

Supplementary Figure 7 | Cryo-EM data processing and model building for *Pd*NorQ^{WB}D^{VWA} with ATP subset 1-3. a, Representative micrograph and 2D classes. **b**, Cryo-EM processing workflow yielding three map subsets. **c**, EM density and atomic model of one NorQ α-helix (aa223-236) in chain B for subset 1-3. **d**, Local resolution map, 3D Fourier shell correlation (FSC) curve and conical FSC (cFSC) curve for subset 1-3.

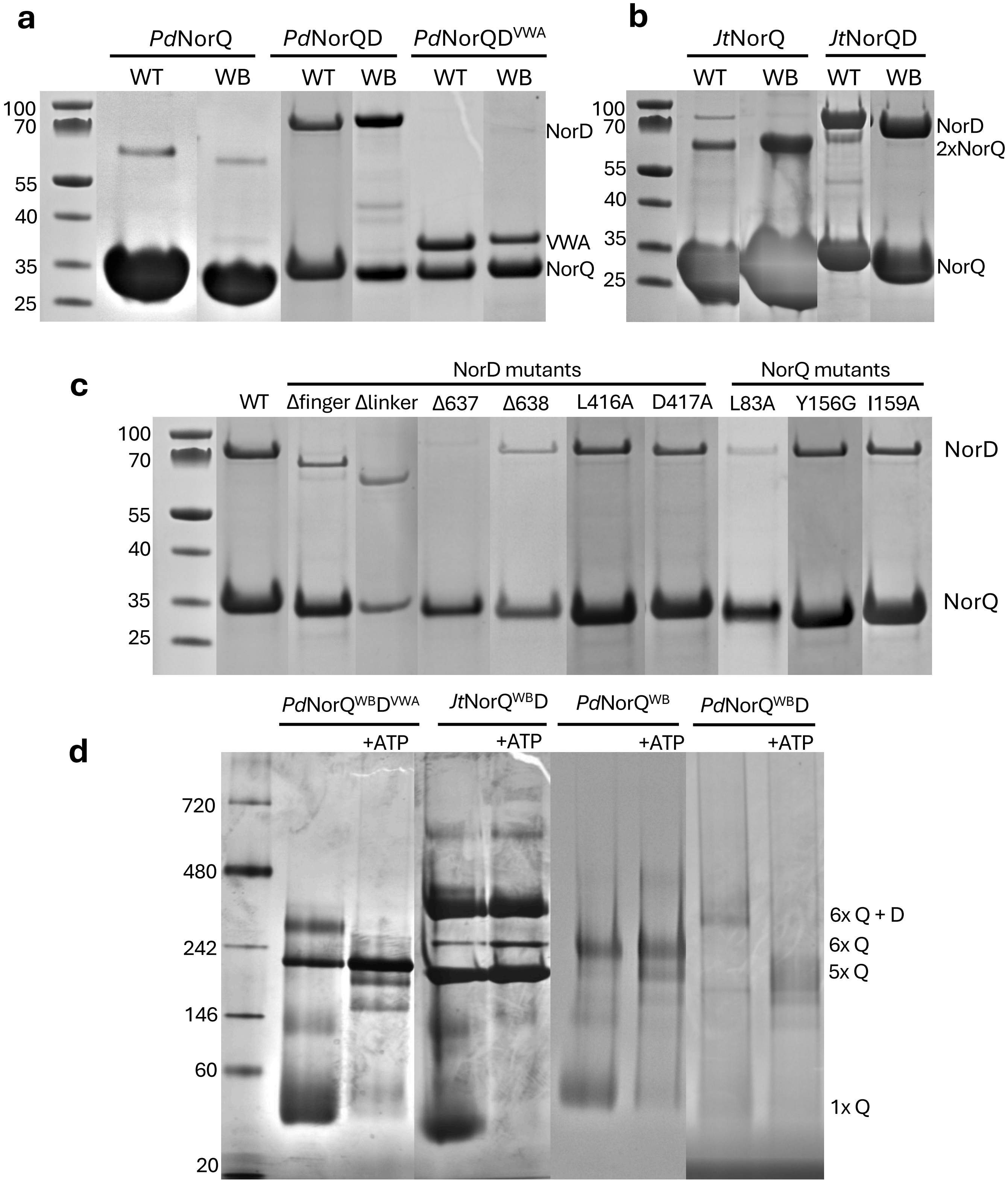

Supplementary Figure 8 | SDS-PAGE and Blue Native gels of purified NorQD proteins. a, SDS-page gels of purified *Pd*NorQD complexes. Lane 1: Ladder. Lane 2: *Pd*NorQ. Lane 3: *Pd*NorQ^{WB}. Lane 4: *Pd*NorQD. Lane 5: *Pd*NorQ^{WB}D. Lane 6: *Pd*NorQD^{VWA}. Lane 7: *Pd*NorQ^{WB}D^{VWA}. **b,** Purified *Jt*NorQD proteins. Lane 1: Ladder. Lane 2: *Jt*NorQ. Lane 3: *Jt*NorQ^{WB}. Lane 4: *Jt*NorQD. Lane 5: *Jt*NorQ^{WB}D. **c,** SDS-page of purified *Pd*NorQD mutants. Lane 1: Ladder. Lane 2: Wild type *Pd*NorQD. Lane 3: *Pd*NorQD with the finger deleted. Lane 4: Deletion of the NorD linker (*Pd*NorD$^{D236\text{-}342}$). Lane 5. Deletion of the two last residues of *Pd*NorD (637 and 638). Lane 6. Deletion of the very last residue of *Pd*NorD (638). Lane 7: Mutation L416A in *Pd*NorD. Lane 8: Mutation D417A in *Pd*NorD. Lane 9: Mutation L83A in *Pd*NorQ. Lane 10: Mutation Y156G in *Pd*NorQ. Lane 11: Mutation I159A in *Pd*NorQ. **d,** Blue Native page of protein complexes. Lane 1: Ladder. Lane 2: *Pd*NorQ^{WB}NorD^{VWA} without nucleotides added. Lane 3: *Pd*NorQ^{WB}D^{VWA} with 2 mM ATP and 15 mM $MgCl_2$. Lane 4: *Jt*NorQ^{WB}D without nucleotides added. Lane 5: *Jt*NorQ^{WB}D with 2 mM ATP and 15 mM $MgCl_2$. Lane 6: *Pd*NorQ^{WB} without nucleotides added. Lane 7: *Pd*NorQ^{WB} with 2 mM ATP and 15 mM $MgCl_2$. Lane 8: *Pd*NorQ^{WB}D without nucleotides added. Lane 9: *Pd*NorQ$_{WB}$D with 2 mM ATP and 15 mM $MgCl_2$. Gel slices for lanes 6-9 from [1] Source data are provided as a Source Data file.

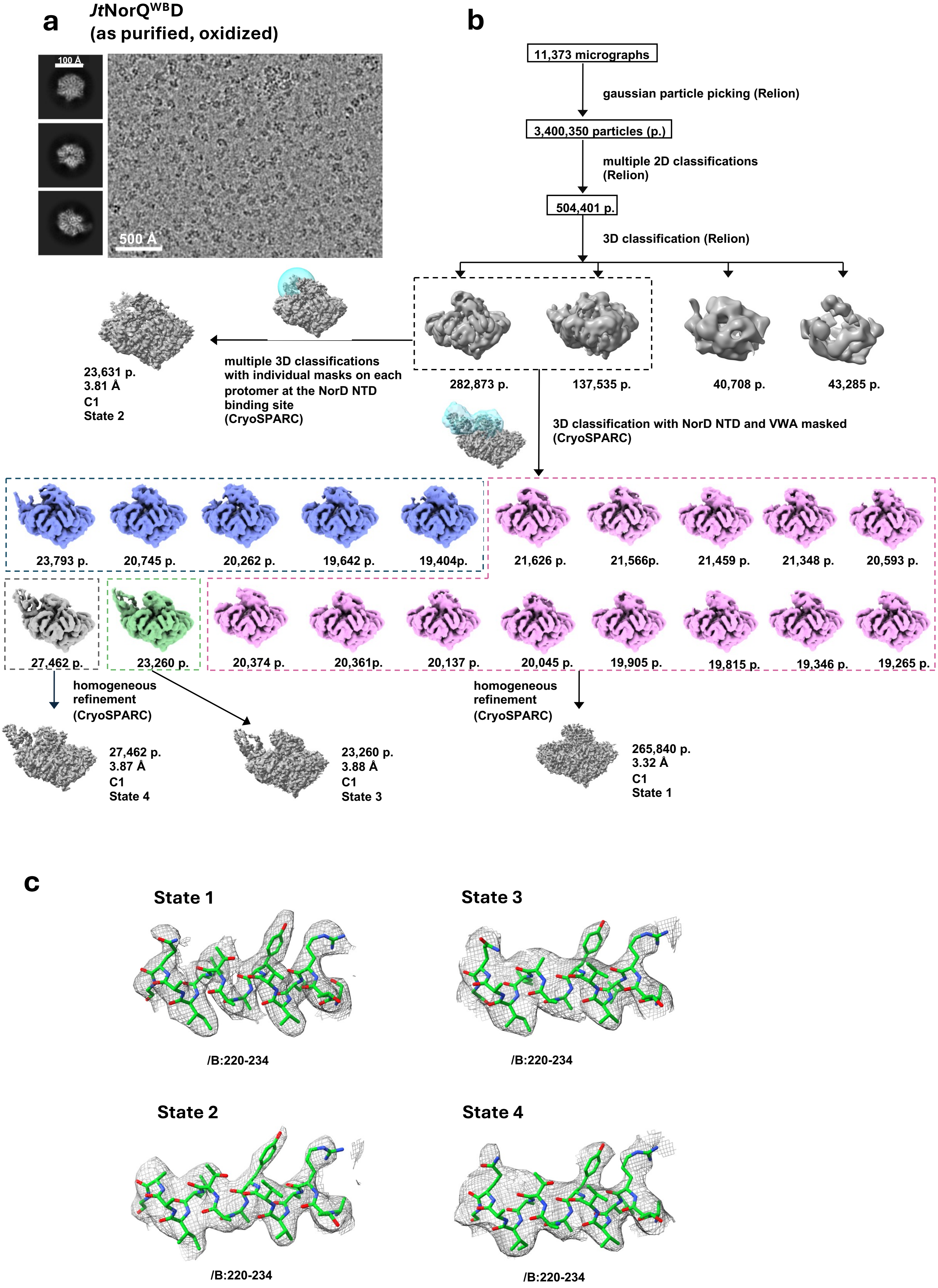
a
JtNorQWBD
(as purified, oxidized)
100 Å
500 Å
b
11,373 micrographs
gaussian particle picking (Relion)
3,400,350 particles (p.)
multiple 2D classifications (Relion)
504,401 p.
3D classification (Relion)
282,873 p.
137,535 p.
40,708 p.
43,285 p.
multiple 3D classifications with individual masks on each protomer at the NorD NTD binding site (CryoSPARC)
23,631 p.
3.81 Å
C1
State 2
3D classification with NorD NTD and VWA masked (CryoSPARC)
23,793 p.
20,745 p.
20,262 p.
19,642 p.
19,404p.
21,626 p.
21,566p.
21,459 p.
21,348 p.
20,593 p.
27,462 p.
23,260 p.
20,374 p.
20,361p.
20,137 p.
20,045 p.
19,905 p.
19,815 p.
19,346 p.
19,265 p.
homogeneous refinement (CryoSPARC)
27,462 p.
3.87 Å
C1
State 4
23,260 p.
3.88 Å
C1
State 3
homogeneous refinement (CryoSPARC)
265,840 p.
3.32 Å
C1
State 1
c
State 1
/B:220-234
State 3
/B:220-234
State 2
/B:220-234
State 4
/B:220-234

Supplementary Figure 9 | Cryo-EM data processing and model building for *Jt*NorQ^{WB}D state 1-4. a, Representative micrograph and 2D classes. **b**, Cryo-EM processing workflow yielding four final map subsets (State 1-4). **c**, EM density and atomic model of one NorQ α-helix (aa223-236) in chain B for state 1-4.

*Jt*NorQ^{WB}D
(as purified, oxidized)

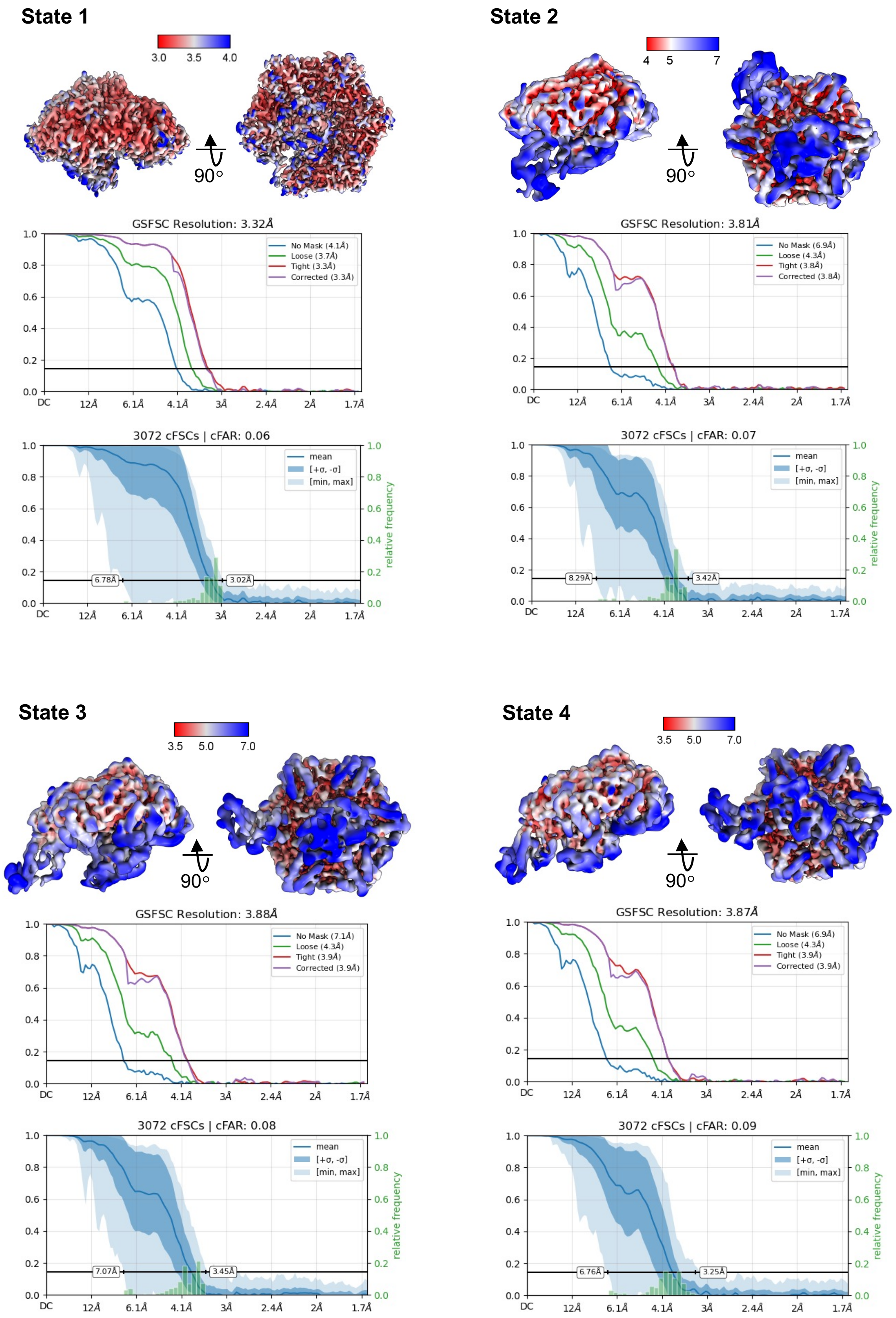

Supplementary Figure 10 | Local resolution map, 3D Fourier shell correlation (FSC) curve and conical FSC (cFSC) curve from processing the *Jt*NorQ^{WB}D dataset for state 1-4.

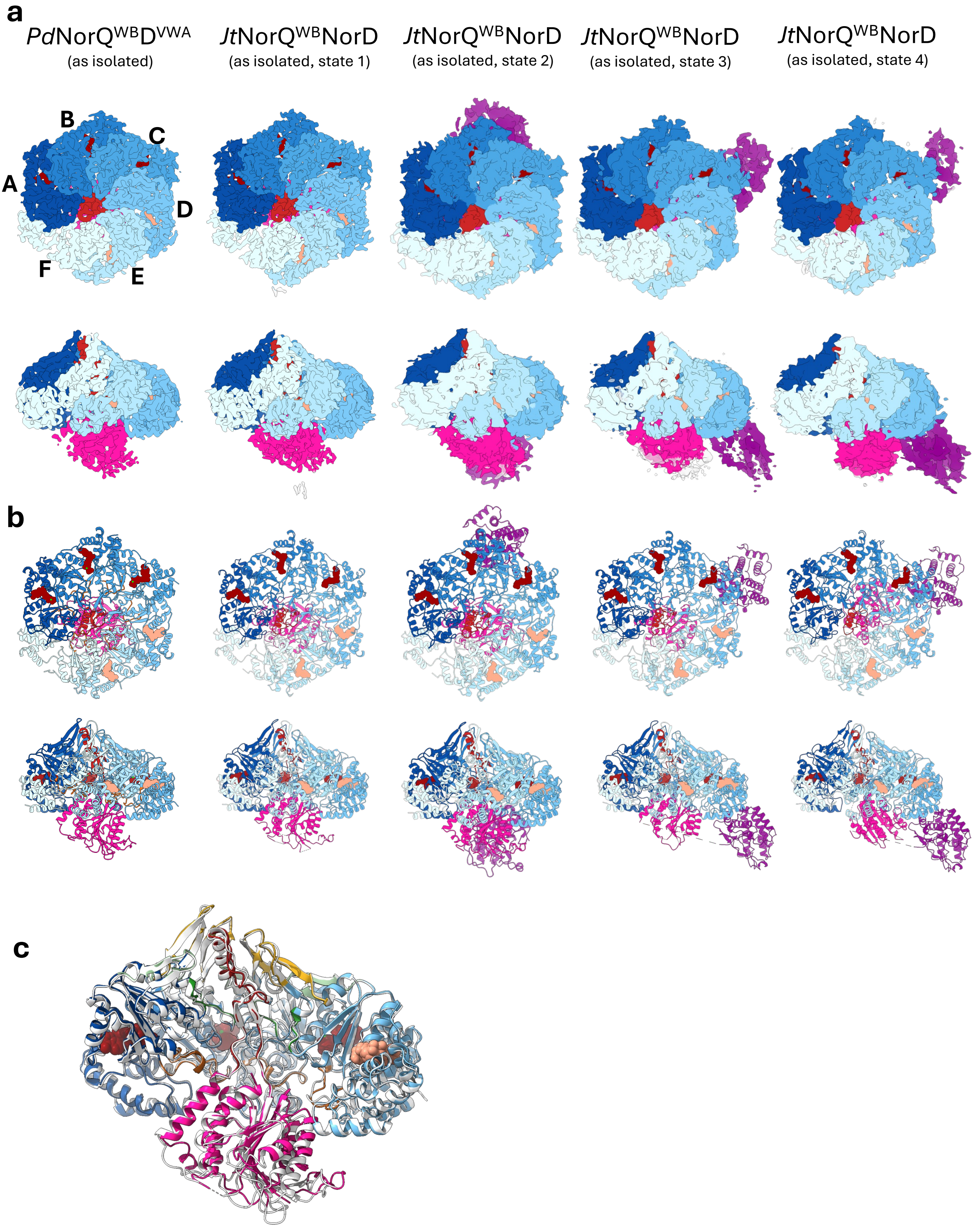

Supplementary Figure 11 | Structures of *Pd*NorQ^{WB}D^{VWA} and *Jt*NorQ^{WB}D. a, Cryo-EM maps of *Pd*NorQ^{WB}D^{VWA} and *Jt*NorQ^{WB}D shown as top view (N-terminal side of NorQ) and side view. The individual subunits of NorQ are colour-coded in shades of blue according to their position in the spiral, from top (dark) to bottom (light). Density for ATP and ADP is shown in dark red and peach, respectively. NorD is colour-coded as shown in Figure 1. All complexes are superimposed on subunit A of NorQ. **b,** Models derived from the maps shown in a. **c,** Superimposition of the oxidized *Pd*NorD^{VWA} model (coloured) and the *Jt*NorQ^{WB}D models (white) on all AAA+ domains. RMSD between 1483 pruned Cα atom pairs is 0.687 Å (across all 1526 Cα atom pairs: 0.953 Å).

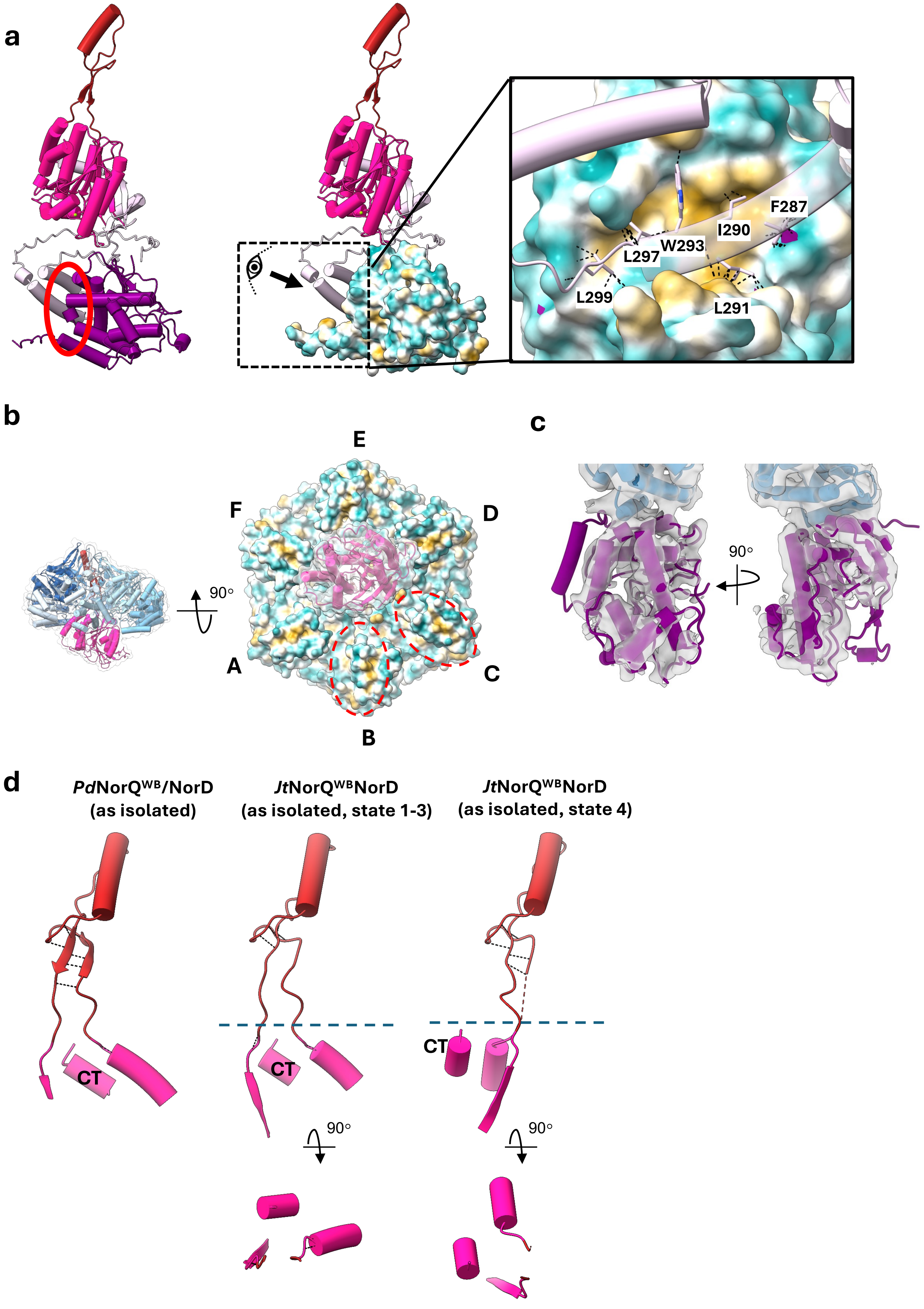
a
F287
I290
W293
L297
L299
L291
b
E
F
D
90°
A
C
B
c
90°
d
PdNorQ^WB/NorD
(as isolated)
JtNorQ^WBNorD
(as isolated, state 1-3)
JtNorQ^WBNorD
(as isolated, state 4)
CT
CT
CT
90°
90°

Supplementary Figure 12 | Structural features of NorD. a, AlphaFold 3 model of *Jt*NorD in the presence of one Mg^{2+} ion (left). The colour code of the ribbon representation is as shown in Figure 1a, The linker sequence (lilac) is predicted with low to very low confidence. The hydrophobic interactions between the NorD N-terminus and the linker region are shown on the right. The NorD N-terminal domain is shown as surface model coloured by hydrophobicity. The inset shows interacting residues of the linker region with a hydrophobic surface patch of the NorD N-terminus. **b,** Surface representation of the *Jt*NorQ^{WB}D model derived from our cryo-EM map as seen from the C-terminal side of the NorQ complex. The NorD surface is depicted transparent and the NorQ surface is coloured by hydrophobicity (right). The binding surface between *Jt*NorQ^{WB} and *Jt*NorD^{NTD} is indicated on NorQ by red, dotted circles. **c,** Cryo EM density (transparent) of *Jt*NorQ^{WB}D (state 4) with rigid body fit of *Jt*NorD N-terminal domain (purple) and *Jt*NorQ (blue). **d,** Ribbon model of finger-motif residues and C-terminal helix of NorD for the specified structures.

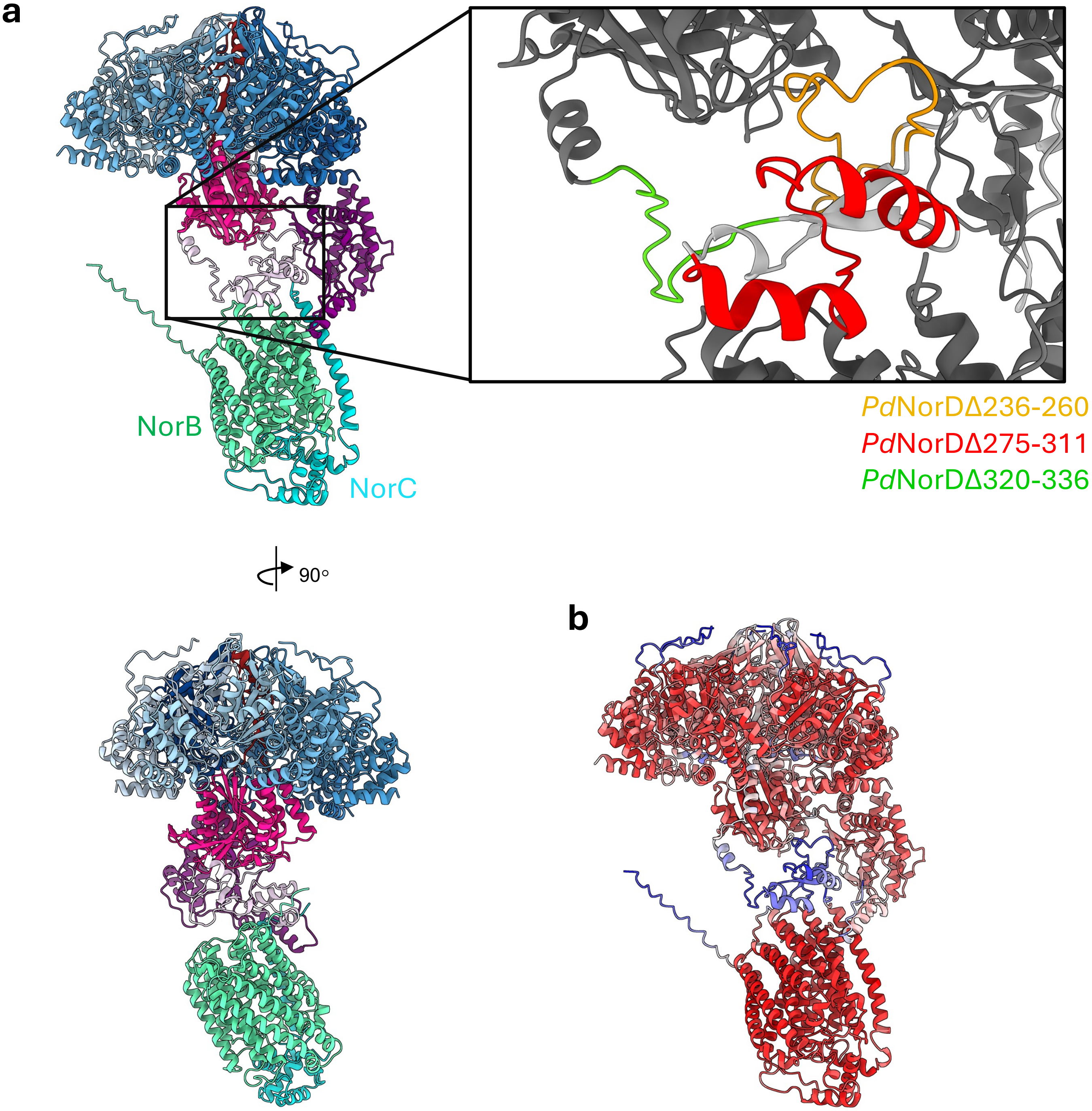

Supplementary Figure 13 | AlphaFold 3 model of the *Pd*NorQ$_6$D$_1$C$_1$B$_1$ complex. a, The colour code of the ribbon representation of NorQD is as shown in Figure 1. NorB is shown in light green and NorC in cyan. The inset shows a close-up of the NorD linker region. The linker deletions used in our functional studies are marked. **b**, The confidence of the prediction is shown as: red=high confidence, white=medium confidence, blue=low confidence. The NorD linker region was modelled with relatively low confidence.

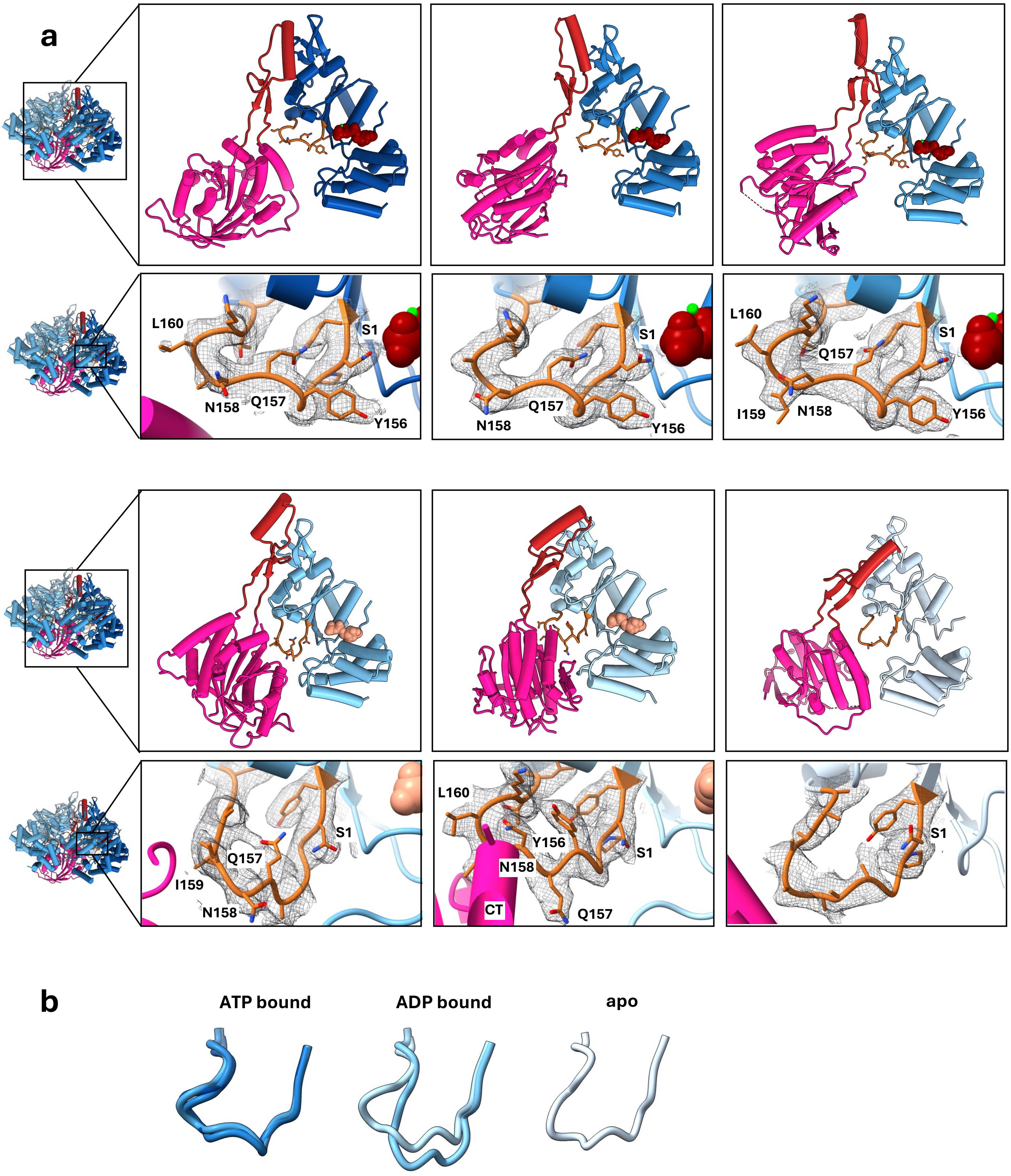

Supplementary Figure 14 | Nucleotide dependent variation in postS1 loop conformation in *Pd*NorQ^{WB}D^{VWA}. The colour code of the ribbon representation is as shown in Figure 1. All NorQ subunits are aligned on the large AAA+ subdomain of chain A. **a**, In the upper row the position of the NorD VWA relative to the respective NorQ protomer is shown. In the lower row a close-up of the respective postS1 loop model and EM density is shown. **b**, Grouped overlays of the postS1 loops in the ATP bound, ADP bound and apo subunit(s).

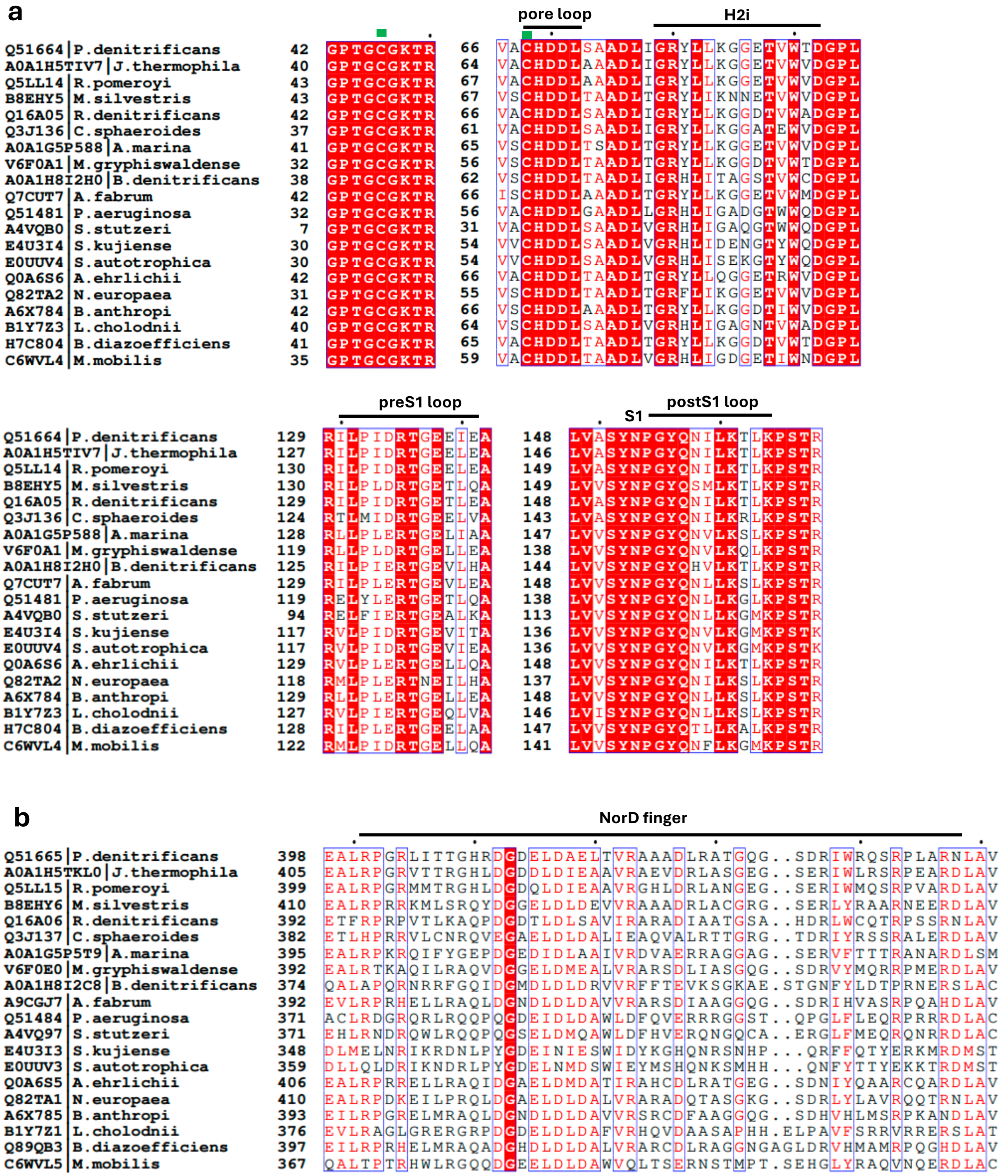

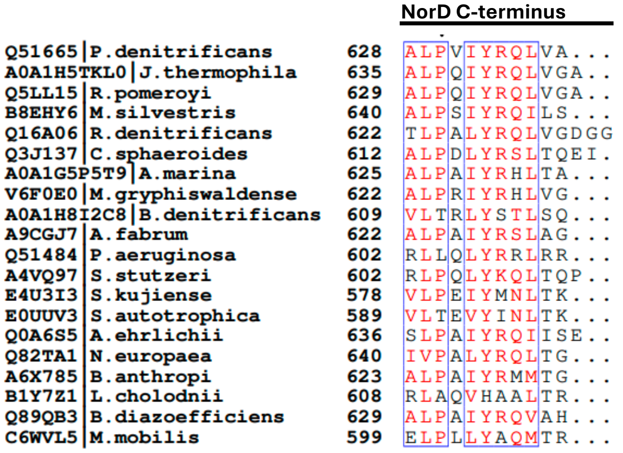

Supplementary Figure 15 | Sequence alignment of NorQ and NorD. The sequences derive from cNOR operons and are named by their Uniprot identifier. Cystein residues involved in the disulfide bridge observed in *Pd*Nor^{QW}B^{VWA} are marked with a green square. **a**, Fragments of the alignment of NorQ showing conservation of the disulfide bridge forming cysteins, pore loop, H2i, preS1 loop and postS1 loop. **b**, Fragments of the alignment of NorD at the VWA domain finger and NorD C-terminus.

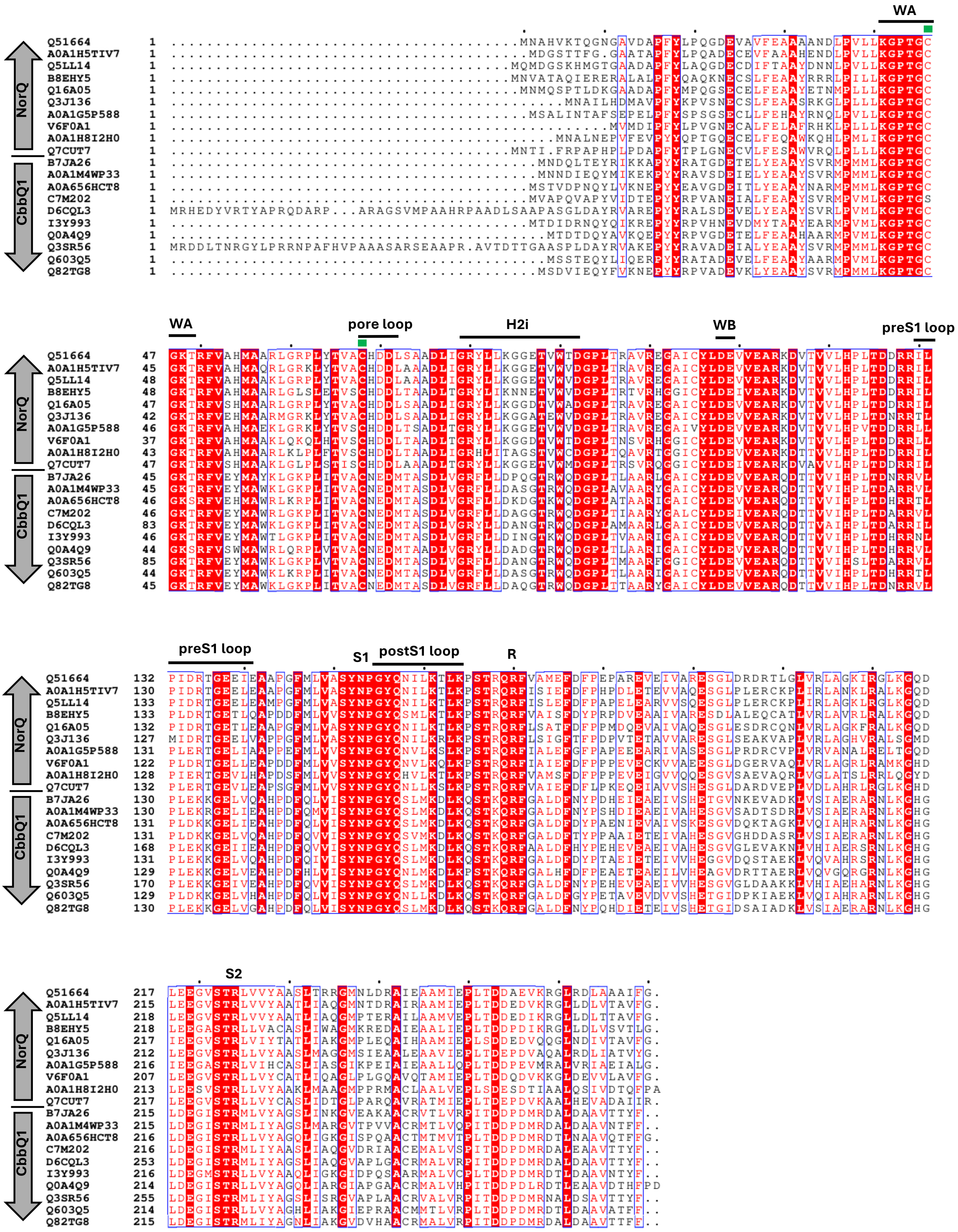

Supplementary Figure 16 | Sequence alignment of NorQ and CbbQ1 with 10 sequences each. All sequences are named by their Uniprot identifier and the disulfide bridge forming cysteins (as observed in *Pd*Nor^{QW}B^{VWA}) are marked with a green square.

Supplementary Tables

	*Pd*NorQ^{WB} (EMDB-53154) (PDB 9QH4)	*Pd*NorQ^{WB} D^{VWA} (EMDB-53156) (PDB 9QH6)	*Pd*NorQ^{WB} D^{VWA}red (EMDB-53158) (PDB 9QH8)	*Pd*NorQ^{WB} D^{VWA}ATP subset 1 (EMDB-53157) (PDB 9QH7)	*Pd*NorQ^{WB} D^{VWA}ATP subset 2 (EMDB-53159) (PDB 9QH9)
**Data collection and processing**					
Magnification	105,000x	105,000x	105,000x	130,000x	130,000x
Voltage (kV)	300	300	300	300	300
Electron exposure (e–/Å^2)	40	45	40	40	40
Defocus range (μm)	0.5-2.5	0.5-2.5	0.5-2.5	0.5-2.5	0.5-2.5
Pixel size (Å)	0.828	0.846	0.828	0.852	0.852
Symmetry imposed	C1	C1	C1	C1	C1
Initial particle images (no.)	4,359,033	3,278,407	4,011,945	4,011,945	4,011,945
Final particle images (no.)	111,934	50,874	51,145	264,861	262,846
Map resolution (Å)	3.7	3.2	3.8	2.9	3.0
FSC threshold	0.143	0.143	0.143	0.143	0.143
Map resolution range (Å)	3.3 - 6.5	3.0 - 5.9	3.4 - 7.7	2.6 - 6.6	2.6 - 7.5
**Refinement**					
Initial model used (PDB code)	AF3 model	AF3 model	AF3 model	AF3 model	AF3 model
Model resolution (Å)	3.7	3.2	3.8	2.9	3.0
FSC threshold	0.143	0.143	0.143	0.143	0.143
Model resolution range (Å)	3.3 - 6.5	3.0 - 5.9	3.4 - 7.7	2.6 - 6.6	2.6 - 7.5
Map sharpening *B* factor (Å^2)	118.0	99.0	138.6	91.5	88.3
Model composition					
Non-hydrogen atoms	10,648	13,496	12,182	10,180	7,965
Protein residues	1,531	1,819	1,785	1,408	1,164
Ligands	ATP: 3<br>ADP: 3	ATP: 3<br>ADP: 2	ATP: 3<br>ADP: 2	MG: 3<br>ATP: 5	MG: 2<br>ATP: 4
B factors (Å^2)					
Protein	138.59/387.52/199.09	48.49/198.32/102.29	160.96/373.05/223.19	72.22/287.12/126.99	80.31/277.44/153.62
Ligand	169.27/242.21/192.96	67.52/101.13/84.26	209.98/228.89/219.50	85.72/144.14/110.25	102.70/186.70/134.37
R.m.s. deviations					
Bond lengths (Å)	0.002	0.003	0.002	0.002	0.002
Bond angles (°)	0.505	0.510	0.492	0.436	0.468
Validation					
MolProbity score	1.63	1.22	1.61	1.19	1.65
Clashscore	3.43	1.12	3.51	2.12	3.56
Poor rotamers (%)	1.66	1.43	1.60	0.90	1.98
Ramachandran plot					
Favored (%)	95.19	95.95	95.54	96.77	95.99
Allowed (%)	4.74	4.05	4.46	3.09	3.83
Disallowed (%)	0.07	0.00	0.00	0.14	0.17

Supplementary Table 1 | Cryo-EM data collection, refinement and validation statistics for *Pd*NorQ^{WB}, *Pd*NorQ^{WB} D^{VWA} , reduced *Pd*NorQ^{WB} D^{VWA} , *Pd*NorQ^{WB} D^{VWA}ATP subset 1, and *Pd*NorQ^{WB} D^{VWA}ATP subset 2.

Sample	Activity (nmol min^{-1} mg^{-1})	Activity (min^{-1} complex^{-1})	Activity (% of purified in DTT)
Purified in DTT	73±13	18±3	100
DTT only in measurement buffer	15 ± 0.6	4 ± 0.2	22
No DTT	0.73 ± 0.2	0.18 ± 0.05	1

Supplementary Table 2 | ATPase activity of *Pd*NorQD purified in 10 mM DTT and with 2 mM DTT added to activity measurement buffer, *Pd*NorQD purified without DTT but with 2 mM DTT in activity measurement buffer and *Pd*NorQD in the complete absence of DTT. Values are given as averages with standard deviation, n=3 (technical replicates). Source data are provided as a Source Data file.

chain	residue	position	donor	chain	residue	position	acceptor	distance (Å)
**/A**	**ARG**	**135**	**NE**	**/G**	**ASP**	**427**	**OD2**	**3.071**
/D	ARG	135	NH1	/G	ASP	414	O	3.592
**/E**	**ARG**	**80**	**NH1**	**/G**	**ASP**	**412**	**OD1**	**2.943**
/E	ARG	135	NH2	/G	ASP	412	OD2	3.322
**/E**	**ASN**	**158**	**ND2**	**/G**	**ALA**	**638**	**O**	**2.955**
**/E**	**ILE**	**159**	**N**	**/G**	**LEU**	**636**	**O**	**3.082**
**/F**	**ARG**	**80**	**NH2**	**/G**	**GLY**	**434**	**O**	**3.011**
**/F**	**LEU**	**83**	**N**	**/G**	**GLY**	**432**	**O**	**2.972**
**/G**	**ARG**	**423**	**NH2**	**/A**	**GLY**	**79**	**O**	**2.979**
**/G**	**ARG**	**429**	**NH2**	**/A**	**GLY**	**86**	**O**	**2.994**
**/G**	**ARG**	**555**	**NH2**	**/D**	**ASN**	**158**	**O**	**2.938**
/G	LEU	618	N	/E	GLY	214	O	3.515
**/G**	**GLN**	**635**	**NE2**	**/E**	**GLN**	**157**	**OE1**	**2.945**

Supplementary Table 3 | Hydrogen bonds between NorQ (chain A-F) and NorD (chain G) in *Pd*NorQ^{WB}D^{VWA} as detected using standard geometric parameters for hydrogen bonds. Hydrogen bonds that are also shown in Figure 2 are highlighted in bold.

NorD				NorQ				
chain	residue 1	position	atom 1	chain	residue 2	position	atom 2	distance (Å)
/G	ASP	412	CG	/E	ARG	80	NH1	3.173
/G	ARG	443	NH1	/E	ARG	114	NH2	2.988
**/G**	**ARG**	**423**	**NH1**	**/A**	**ARG**	**80**	**NH1**	**3.004**
/G	ALA	638	C	/E	ASN	158	ND2	3.265
**/G**	**ALA**	**418**	**CB**	**/B**	**ARG**	**80**	**NH1**	**3.336**
**/G**	**GLN**	**433**	**CB**	**/F**	**ARG**	**80**	**NH2**	**3.342**
/G	ARG	423	NH2	/A	ALA	75	CB	3.363
/G	ARG	589	CD	/D	GLN	215	O	3.176
**/G**	**ALA**	**425**	**CB**	**/B**	**LEU**	**83**	**CD1**	**3.728**
/G	ALA	399	CB	/F	ILE	159	CB	3.733
/G	VAL	422	CG1	/B	THR	88	CG2	3.785
/G	LEU	636	O	/E	ILE	159	CG1	3.334
**/G**	**ALA**	**430**	**CB**	**/A**	**TYR**	**81**	**CE1**	**3.675**
**/G**	**GLY**	**432**	**O**	**/F**	**LEU**	**83**	**CB**	**3.339**
/G	VAL	637	CG2	/E	LEU	160	CD1	3.812
**/G**	**ALA**	**430**	**CB**	**/A**	**TYR**	**81**	**CD1**	**3.734**
**/G**	**GLU**	**419**	**CD**	**/A**	**ARG**	**80**	**NH2**	**3.617**
/G	LEU	618	CD1	/E	GLY	214	O	3.408
/G	ALA	638	CA	/E	ASN	158	ND2	3.63
/G	ARG	423	CZ	/A	ALA	75	CB	3.615
/G	SER	552	CA	/D	ILE	159	CG2	3.898
**/G**	**GLU**	**419**	**OE2**	**/A**	**ARG**	**80**	**NH2**	**2.824**
**/G**	**GLY**	**434**	**C**	**/E**	**LEU**	**83**	**CD2**	**3.664**
/G	VAL	637	CG2	/E	LEU	160	CD2	3.95
**/G**	**GLY**	**434**	**CA**	**/E**	**LEU**	**83**	**CD2**	**3.952**
**/G**	**GLY**	**432**	**O**	**/F**	**LEU**	**83**	**CA**	**3.504**
/G	ALA	638	N	/E	ASN	158	ND2	3.485
**/G**	**SER**	**435**	**N**	**/E**	**LEU**	**83**	**CD2**	**3.727**
/G	GLY	591	CA	/D	ASN	158	ND2	3.745
/G	LEU	556	CD1	/E	ILE	159	CD1	3.999
**/G**	**LEU**	**416**	**CD1**	**/D**	**THR**	**88**	**CG2**	**4.005**
/G	ARG	443	NH1	/E	ARG	114	NE	3.534
/G	LEU	618	CB	/E	GLY	214	O	3.56
**/G**	**ARG**	**411**	**NH1**	**/E**	**LEU**	**82**	**CD2**	**3.789**
/G	ARG	395	O	/A	ILE	159	CB	3.576
/G	ARG	555	NH2	/D	ASN	158	O	2.938
**/G**	**ALA**	**430**	**CB**	**/A**	**TYR**	**81**	**CZ**	**3.769**
**/G**	**ALA**	**430**	**CB**	**/A**	**TYR**	**81**	**CE2**	**3.922**
/G	ALA	399	CB	/A	LEU	160	CD2	4.043
/G	ASP	412	OD1	/E	ARG	80	NH1	2.943
/G	VAL	422	CG2	/B	TYR	81	O	3.585
/G	GLN	635	NE2	/E	GLN	157	OE1	2.945
**/G**	**ALA**	**426**	**CB**	**/A**	**TYR**	**81**	**O**	**3.586**
/G	ALA	638	O	/E	ASN	158	ND2	2.955
**/G**	**GLN**	**433**	**CG**	**/F**	**ARG**	**80**	**NH2**	**3.821**
**/G**	**ARG**	**429**	**CB**	**/A**	**THR**	**88**	**CG2**	**4.066**
**/G**	**ARG**	**423**	**NH1**	**/A**	**ARG**	**80**	**CZ**	**3.558**
/G	GLY	614	O	/E	GLN	157	NE2	2.968
**/G**	**GLY**	**432**	**O**	**/F**	**LEU**	**83**	**N**	**2.972**
**/G**	**ARG**	**423**	**NH2**	**/A**	**GLY**	**79**	**O**	**2.979**
/G	ALA	593	CB	/E	ILE	159	CG2	4.079
**/G**	**ALA**	**426**	**CB**	**/A**	**TYR**	**81**	**CB**	**4.088**
**/G**	**ARG**	**429**	**CB**	**/A**	**LEU**	**83**	**CD2**	**4.091**
**/G**	**ARG**	**429**	**NH2**	**/A**	**GLY**	**86**	**O**	**2.994**
**/G**	**ALA**	**430**	**CB**	**/A**	**TYR**	**81**	**CD2**	**3.976**
/G	LEU	405	CD2	/C	ARG	114	NH1	3.859
**/G**	**GLY**	**434**	**O**	**/F**	**ARG**	**80**	**NH2**	**3.011**
**/G**	**ALA**	**430**	**CB**	**/A**	**THR**	**88**	**CG2**	**4.112**
/G	LEU	636	O	/E	ASN	158	CA	3.655
/G	LEU	405	CD2	/C	ARG	114	CZ	3.853
**/G**	**ASP**	**427**	**CG**	**/A**	**ARG**	**135**	**NE**	**3.889**
/G	ARG	404	NH1	/A	ASP	70	CG	3.894
**/G**	**ALA**	**430**	**CB**	**/A**	**THR**	**88**	**CB**	**4.138**
/G	ALA	551	CB	/D	LEU	160	CB	4.139
/G	LEU	618	CD1	/E	GLY	214	CA	4.15
**/G**	**LEU**	**416**	**CD1**	**/C**	**LEU**	**83**	**CB**	**4.152**

Supplementary Table 4 | Contacts between NorQ and NorD in *Pd*NorQ^{WB}D^{VWA} as detected using interaction parameters for atom pairs with VDW overlap of ≥ -0.4 Å. Contacts that are also shown in Figure 2 are highlighted in bold.

Sample	Activity (nmol min^{-1} mg^{-1})	Activity (min^{-1} $complex^{-1}$)	% of WT
P. denitrificans			
*Pd*hQ	76 ± 7	13±1	100
*Pd*hQ^{WB}	0.6±0.1	0.1±0.02	0.8
*Pd*Qs	66 ±12	12±2	100
*Pd*QD	73±13	18±3	100
*Pd*Q^{WB}D	0.4 ± 0.05	0.1 ± 0.01	0.6
*Pd*QD^{VWA}	49±3	10±0.7	56
*Pd*Q^{WB}D^{VWA}	0.4±0.03	0.08±0.006	0.4
J. thermophila			
*Jt*Q	80±20	15±3	100
*Jt*QD	59±8	15±2	100
*Jt*Q^{WB}D	0.97±0.2	0.24±0.05	1.6
**Mutants**			
*Pd*QD$^{\Delta finger}$	110±10	28±3	155
*Pd*QD$^{\Delta 638}$	61±5	15±1	83
*Pd*Q^{Y156G}D	85±10	21±3	120
*Pd*Q^{I159A}D	65±16	16 ±4	89
*Pd*QD^{L416A}	52±9	13±2	72
*Pd*QD^{D417A}	38±3	10±0.6	56

Supplementary Table 5 | ATPase activities of NorQD protein complexes purified in the presence of 10 mM DTT. Activity was measured using the malachite green end-point assay. Protein concentration 0.025-0.06 mg/mL. Values are given as averages with standard deviation, *n*=3 (technical replicates). Source data are provided as a Source Data file.

Sample	Activity (% of WT)	Fe_B content (% of WT)
*Pd-c*NOR[a]	100[a]	100[a]
*Pd-c*NOR-ΔNorQD[a]	0.4[a]	20 ± 7[a]
*Pd-c*NOR-Δfinger	1.4 ± 0.3	14 ± 7

Supplementary Table 6 | NO-reduction activity and Fe_B content of *Pd-c*NOR expressed with and without NorQD, and without the NorD finger. Values are given as averages with standard deviation, *n*=2 (technical replicates). [a]Data from [2]. Source data are provided as a Source Data file.

	*Jt*NorQ^{WB}D state 1 (EMDB-53160) (PDB 9QHD)	*Jt*NorQ^{WB}D state 2 (EMDB-53161) (PDB 9QHE)	*Jt*NorQ^{WB}D state 3 (EMDB-53162) (PDB 9QHF)	*Jt*NorQ^{WB}D state 4 (EMDB-53163) (PDB 9QHG)
**Data collection and processing**				
Magnification	105,000x	105,000x	105,000x	105,000x
Voltage (kV)	300	300	300	300
Electron exposure (e–/Å^2)	45	45	45	45
Defocus range (μm)	0.5-2.5	0.5-2.5	0.5-2.5	0.5-2.5
Pixel size (Å)	0.846	0.846	0.846	0.846
Symmetry imposed	C1	C1	C1	C1
Initial particle images (no.)	3,400,350	3,400,350	3,400,350	3,400,350
Final particle images (no.)	265,840	23,631	23,260	27,462
Map resolution (Å)	3.3	3.8	3.9	3.9
FSC threshold	0.143	0.143	0.143	0.143
Map resolution range (Å)	3.0 - 6.1	3.5 - 12.1	3.6 - 10.5	3.5 - 11.9
**Refinement**				
Initial model used (PDB code)	AF3 model	AF3 model	AF3 model	AF3 model
Model resolution (Å)	3.3	3.8	3.9	3.9
FSC threshold	0.143	0.143	0.143	0.143
Model resolution range (Å)	3.0 - 6.1	3.5 - 12.1	3.6 - 10.5	3.5 - 11.9
Map sharpening *B* factor (Å^2)	135.0	79.4	78.7	79.9
Model composition				
Non-hydrogen atoms	13,295	11,435	10,853	11,094
Protein residues	1,791	1,764	1,728	1,748
Ligands	ATP: 3<br>ADP: 2	ATP: 3<br>ADP: 2	ATP: 3<br>ADP: 2	ATP: 3<br>ADP: 2
B factors (Å^2)				
Protein	44.62/184.91/106.00	36.92/226.48/92.16	91.21/358.35/171.06	101.42/432.05/171.03
Ligand	71.32/123.51/93.96	76.43/93.67/82.67	162.59/209.33/178.51	138.76/205.61/168.18
R.m.s. deviations				
Bond lengths (Å)	0.002	0.006	0.002	0.003
Bond angles (°)	0.518	0.636	0.473	0.548
Validation				
MolProbity score	1.66	2.02	1.41	1.65
Clashscore	2.74	3.88	2.42	3.65
Poor rotamers (%)	2.14	3.61	0.34	0.16
Ramachandran plot				
Favored (%)	95.03	93.92	94.24	91.70
Allowed (%)	4.97	6.71	5.70	8.13
Disallowed (%)	0.00	0.00	0.06	0.17

Supplementary Table 7 | Cryo-EM data collection, refinement and validation statistics for *Jt*NorQ^{WB}D state 1, *Jt*NorQ^{WB}D state 2, *Jt*NorQ^{WB}D state 3 and *Jt*NorQ^{WB}D state 4.

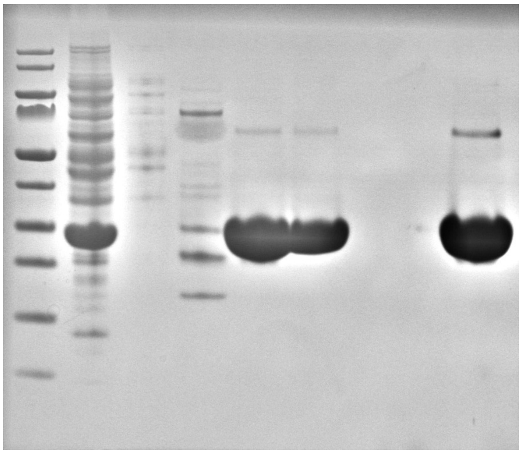

Fig S8a) lane 2

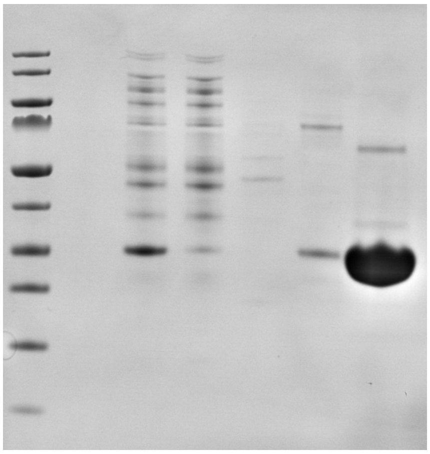

Fig S8a) lane 3

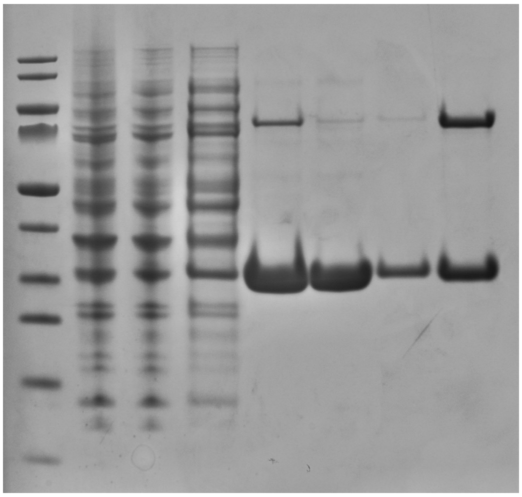

Fig S8a) lane 4, Fig S8c) lane 2 and Fig 2e) lane 1

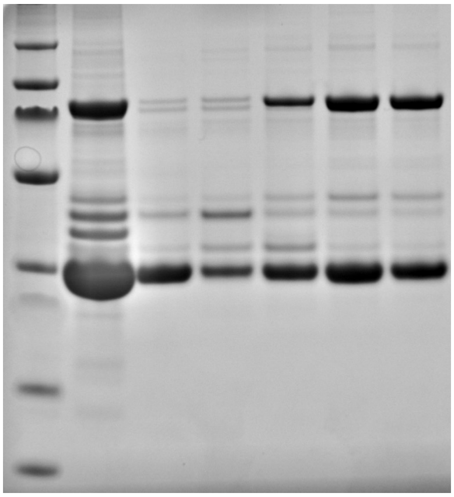

Fig S8a) lane 5

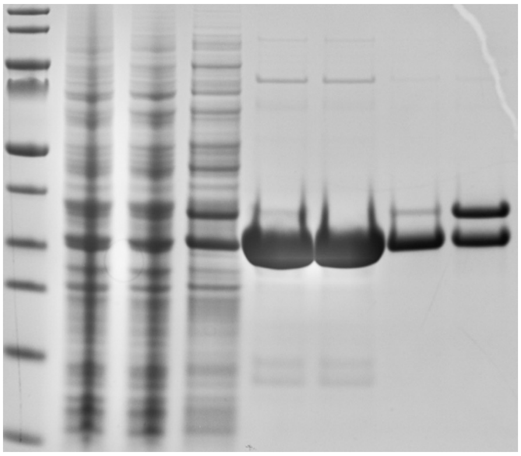

Fig S8a) lane 6

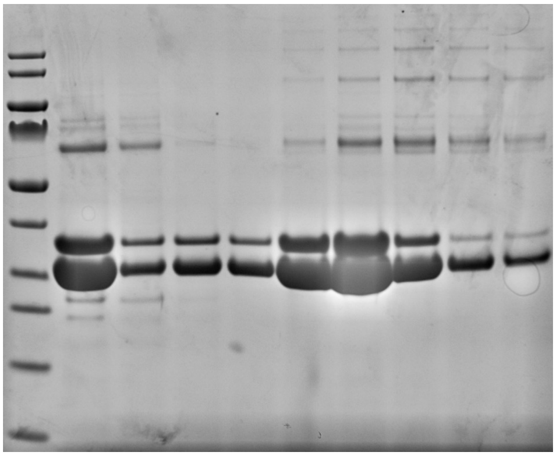

Fig S8a) lane 7

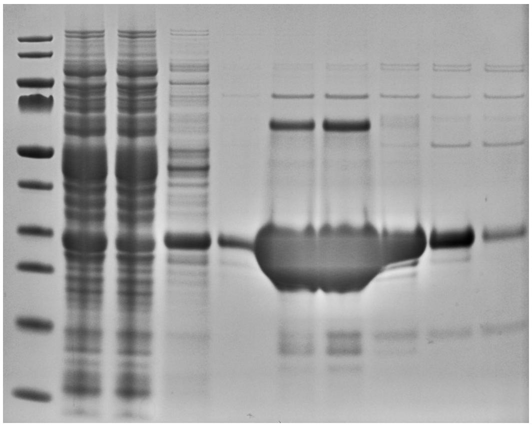

Fig S8b) lane 2

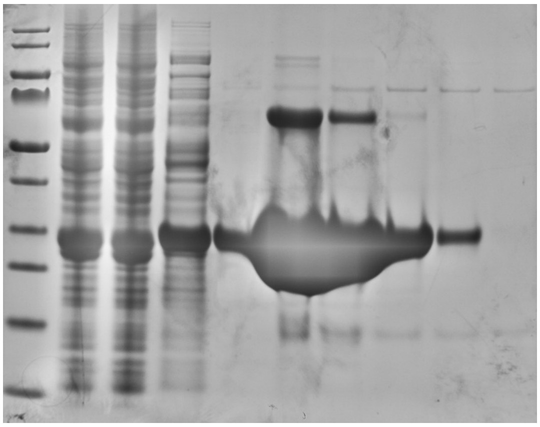

Fig S8b) lane 3

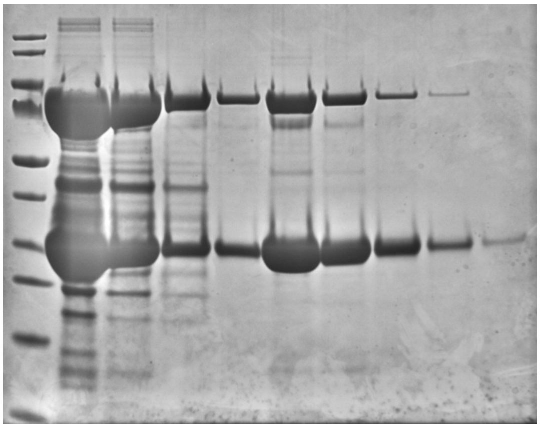

Fig S8b) lane 4

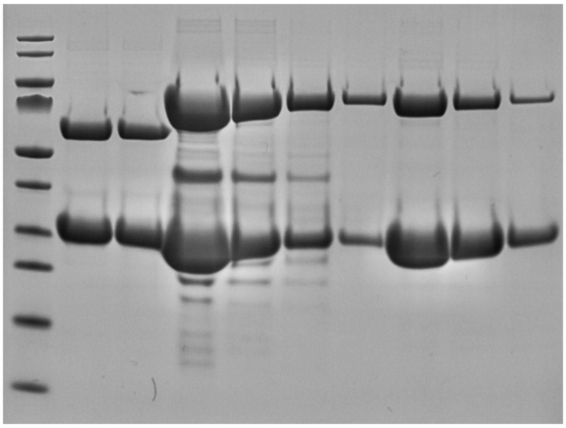

Fig S8b) lane 5

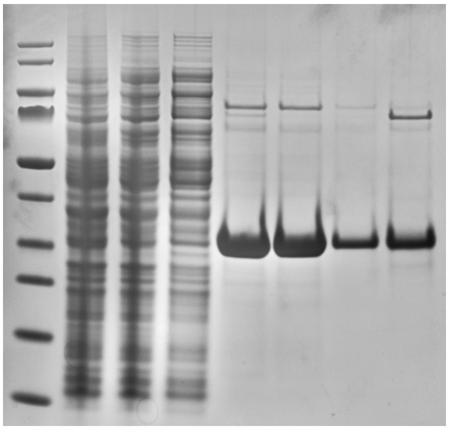

Fig S8c) lane 3 and
Fig 2e) lane 2

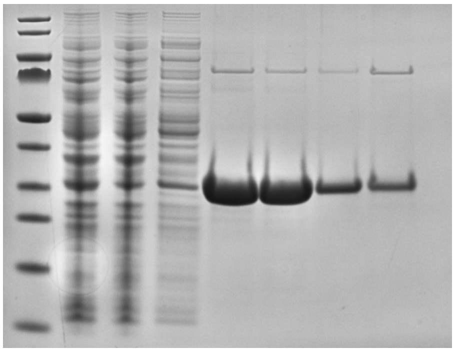

Fig S8c) lane 6

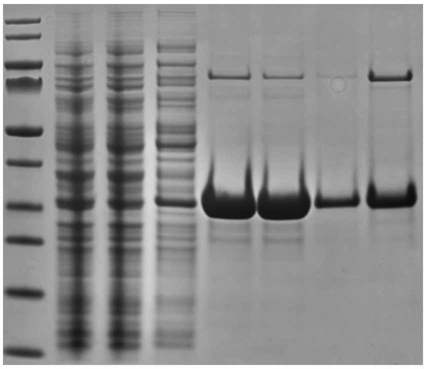

Fig S8c) lane 7

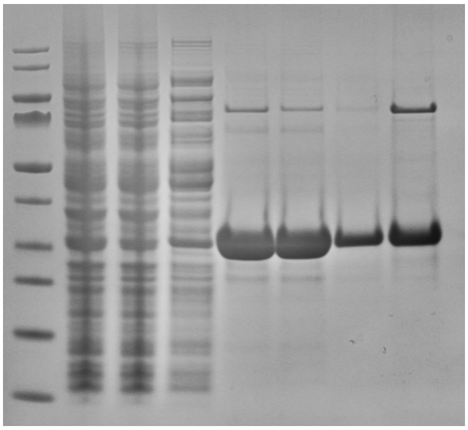

Fig S8c) lane 8

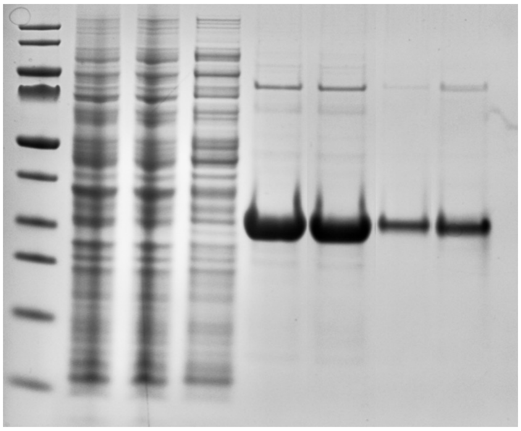

Fig S8c) lane 9

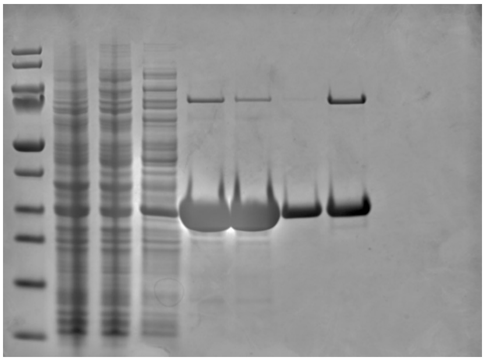

Fig S8c) lane 10

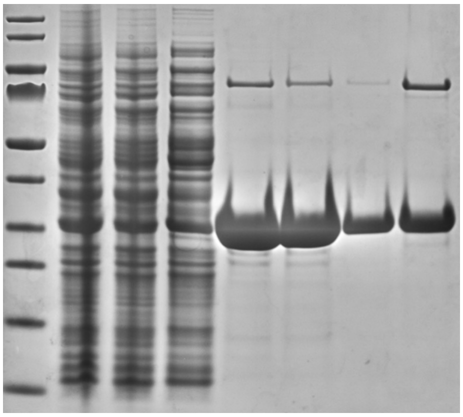

Fig S8c) lane 11

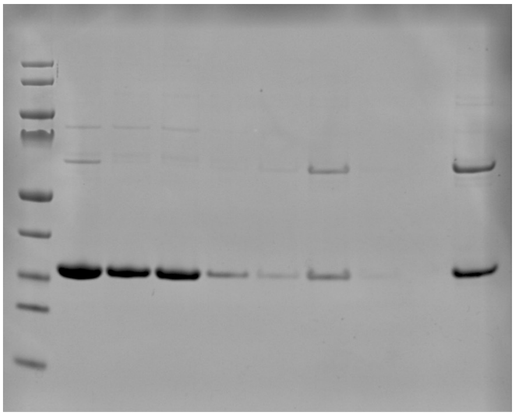

Fig S8c) lane 4

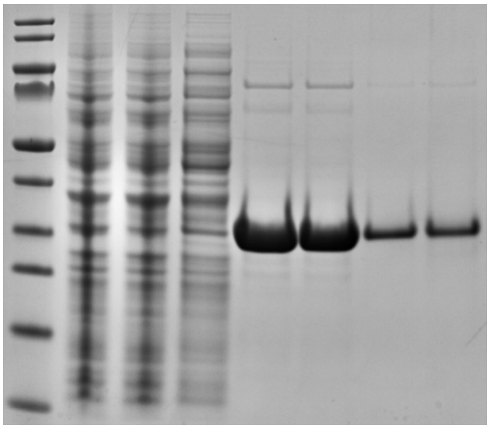

Fig S8c) lane 5 and Fig 2e) lane 3

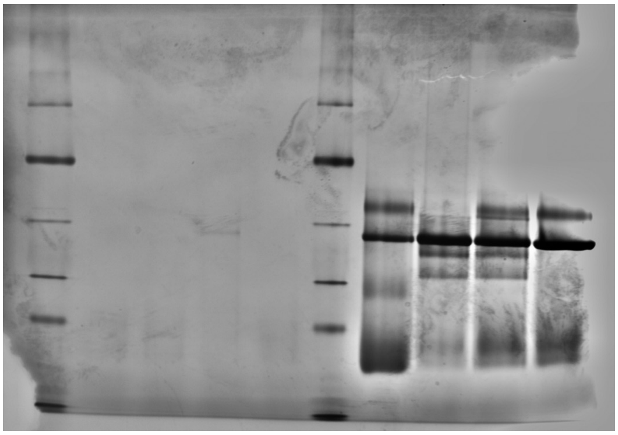

Fig S8d) lanes 2 and 3

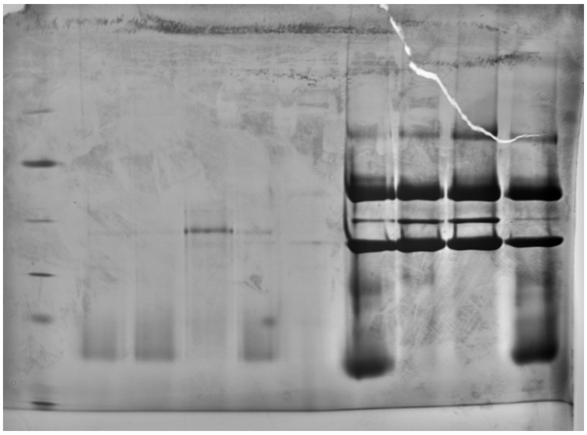

Fig S8d) lanes 4 and 5

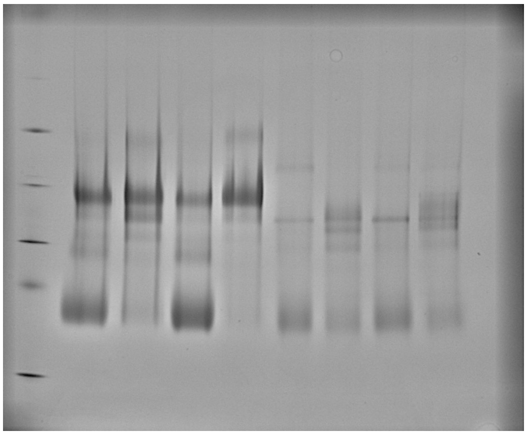

Fig S8d) lanes 6 and 7

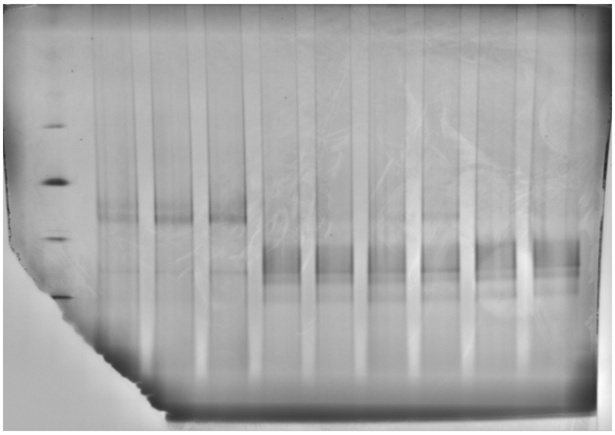

Fig S8d) lanes 8 and 9

References

1. Kahle, M., et al., *Insights into the structure-function relationship of the NorQ/NorD chaperones from Paracoccus denitrificans reveal shared principles of interacting MoxR AAA+/VWA domain proteins*. BMC biology, 2023. **21**(1): p. 47.

2. Kahle, M., et al., *The insertion of the non-heme Fe_B cofactor into nitric oxide reductase from P. denitrificans depends on NorQ and NorD accessory proteins*. Biochimica et Biophysica Acta (BBA)-Bioenergetics, 2018. **1859**(10): p. 1051-1058.
