# Supplementary material for: NorQD AAA+ complex drives metal insertion by a twisting mechanism
Source: Nat Commun. 2026 Mar 27;17:3032. doi: 10.1038/s41467-026-71044-4 (PMC13036017; doi:10.1038/s41467-026-71044-4)
Supplement: Supplementary file 2 — Description of Additional Supplementary Files [file 41467_2026_71044_MOESM2_ESM.pdf]

Description of Additional Supplementary Files

File name: Supplementary Movie 1
Description: Tilting motion of the NorD VWA domain in *Pd*NorQ^{WB}D^{VWA} from multi-body refinement in Relion. Two rigid bodies were defined by masks around 1) the NorQ hexameric ring and 2) the NorD VWA domain.

File name: Supplementary Movie 2
Description: Structural transitions from State 2-4 in *Jt*NorQ^{WB}D. Shown is the surface representation of the atomic models with the colour coding of subunits/domains as shown in Figure 1. The transitions from State 2 over State 3 to State 4 are first shown from the NorQ N-terminal side. The sequence is then repeated viewed from the C-terminus of NorQ and from the side of the NorQ ring.
